# Apolipoprotein A-I anti-tumor activity targets cancer cell metabolism

**DOI:** 10.18632/oncotarget.27590

**Published:** 2020-05-12

**Authors:** Maryam Zamanian-Daryoush, Daniel J. Lindner, Jennifer Buffa, Banu Gopalan, Jie Na, Stanley L. Hazen, Joseph A. DiDonato

**Affiliations:** ^1^Department of Cardiovascular & Metabolic Sciences, Lerner Research Institute, Cleveland Clinic, Cleveland, OH 44195, USA; ^2^Taussig Cancer Institute, Cleveland Clinic, Cleveland Clinic, Cleveland, OH 44195, USA; ^3^Yorg Corporation, Plano, TX 75093, USA; ^4^Department of Health Science Research, Mayo Clinic, Rochester, MN 55905, USA; ^5^Department of Cardiovascular Medicine, Heart and Vascular Institute, Cleveland Clinic, Cleveland, OH 44195, USA

**Keywords:** cancer, apolipoprotein A-I, cholesterol, mevalonate pathway, *de novo* serine synthesis pathway

## Abstract

Previously, we reported apolipoprotein A-I (apoA-I), the major protein component of high-density lipoprotein (HDL), has potent anti-melanoma activity. We used DNA microarray and bioinformatics to interrogate gene expression profiles of tumors from apoA-I expressing (A-I Tg^+/–^) versus apoA-I-null (A-I KO) animals to gain insights into mechanisms of apoA-I tumor protection. Differential expression analyses of 11 distinct tumors per group with > 1.2-fold cut-off and a false discovery rate adjusted *p* < 0.05, identified 176 significant transcripts (71 upregulated and 105 downregulated in A-I Tg^+/–^ versus A-I KO group). Bioinformatic analyses identified the mevalonate and *de novo* serine/glycine synthesis pathways as potential targets for apoA-I anti-tumor activity. Relative to A-I KO, day 7 B16F10L melanoma tumor homografts from A-I Tg^+/–^ exhibited reduced expression of mevalonate-5-pyrophosphate decarboxylase (*Mvd*), a key enzyme targeted in cancer therapy, along with a number of key genes in the sterol synthesis arm of the mevalonate pathway. Phosphoglycerate dehydrogenase (*Phgdh*), the first enzyme branching off glycolysis into the *de novo* serine synthesis pathway, was the most repressed transcript in tumors from A-I Tg^+/–^. We validated our mouse tumor studies by comparing the significant transcripts with adverse tumor markers previously identified in human melanoma and found 45% concordance. Our findings suggest apoA-I targets the mevalonate and serine synthesis pathways in melanoma cells *in vivo*, thus providing anti-tumor metabolic effects by inhibiting the flux of biomolecular building blocks for macromolecule synthesis that drive rapid tumor growth.

## INTRODUCTION

Melanoma is a cancer derived from melanocytes, the pigment-producing cells of epidermis and hair follicles. It is trending upward in incidence and mortality worldwide, and has no effective treatment post metastasis [1–3]. This complex disease is driven by both genetic and epigenetic factors, and many studies have focused on gaining a more thorough understanding of the molecular mechanisms involved in disease progression [4]. Gene expression profiling of primary [5–8] or metastatic tumor specimens, as well as cell lines [6, 9–12], have identified disease markers and gene signatures for different stages of melanoma.

High-density lipoprotein (HDL), a physiological plasma molecule long known for its atheroprotective properties [13, 14], was linked to cancer in a large meta-analysis of randomized controlled trials of lipid-altering therapies that suggested an inverse relationship between plasma HDL cholesterol (HDL-c) levels and incident development of cancer [15]. Previously, we reported a potent anti-tumorigenic activity for apolipoprotein A-I (apoA-I), the major protein component of HDL, against human melanoma A375 and the highly aggressive and metastatic mouse melanoma B16F10L [16]. Accordingly, growth of syngeneic B16F10L tumor cells was severely restricted in animals expressing human apoA-I (A-ITg^+/–^; high plasma HDL-c levels) relative to apoA-I null mice (A-I KO; low plasma HDL-c levels). Importantly, apoA-I therapy curbed further growth of established tumors in A-I KO and induced tumor regression, thus preventing metastases and prolonging survival [16]. Tumor inhibition by apoA-I was also observed with human melanoma A375 in nude mice. We proposed an immunomodulatory role for apoA-I in melanoma with both innate and adaptive arms of immunity mediating its anti-tumor activity [16].

Cancer cells have different metabolic requirements from normal quiescent cells, given the propensity of malignant cells to proliferate at a high rate and avoid apoptotic death signals. To fulfill these priorities, cancer cells hijack normal metabolic and signaling pathways and redirect them to meet their increased need for biomolecules to synthesize proteins, lipids, and nucleic acids [17–22]. Altered cellular metabolism is now widely considered a hallmark of cancer, and interventions to disrupt cancer metabolism are fast emerging as viable therapeutic approaches in conjunction with conventional death-inducing chemotherapies [23–27]. Statins, a family of lipid-lowering drugs that target 3-hydroxy-3-methyl-glutaryl-co-enzyme A reductase (HMG-CoA reductase), the rate-limiting enzyme at the core of the mevalonate biochemical pathway, have been a focal point of research in the cancer field because their action leads to reduced cholesterol and other key metabolic end products such as activated (prenylated) small GTPases with oncogenic activity [24, 28–31]. Statins have been associated with reduced mortality from several cancers including prostate, kidney, colorectal, breast, and lung cancer [32–39]. Meta-analyses have also suggested a positive correlation between statin use and reduced incidence of melanoma [30].

Herein, we used differential gene expression analysis of primary B16F10L melanoma homografts to investigate the role of host apoA-I in the tumor microenvironment, and identified the mevalonate and *de novo* serine synthesis metabolic pathways as potential targets of apoA-I anti-tumor activity.

## RESULTS

### Whole-genome expression profiling and hierarchical clustering discriminate apoA-I-null-mouse-derived tumors from less aggressive tumors in apoA-I-expressing mice

**Figure 1:**
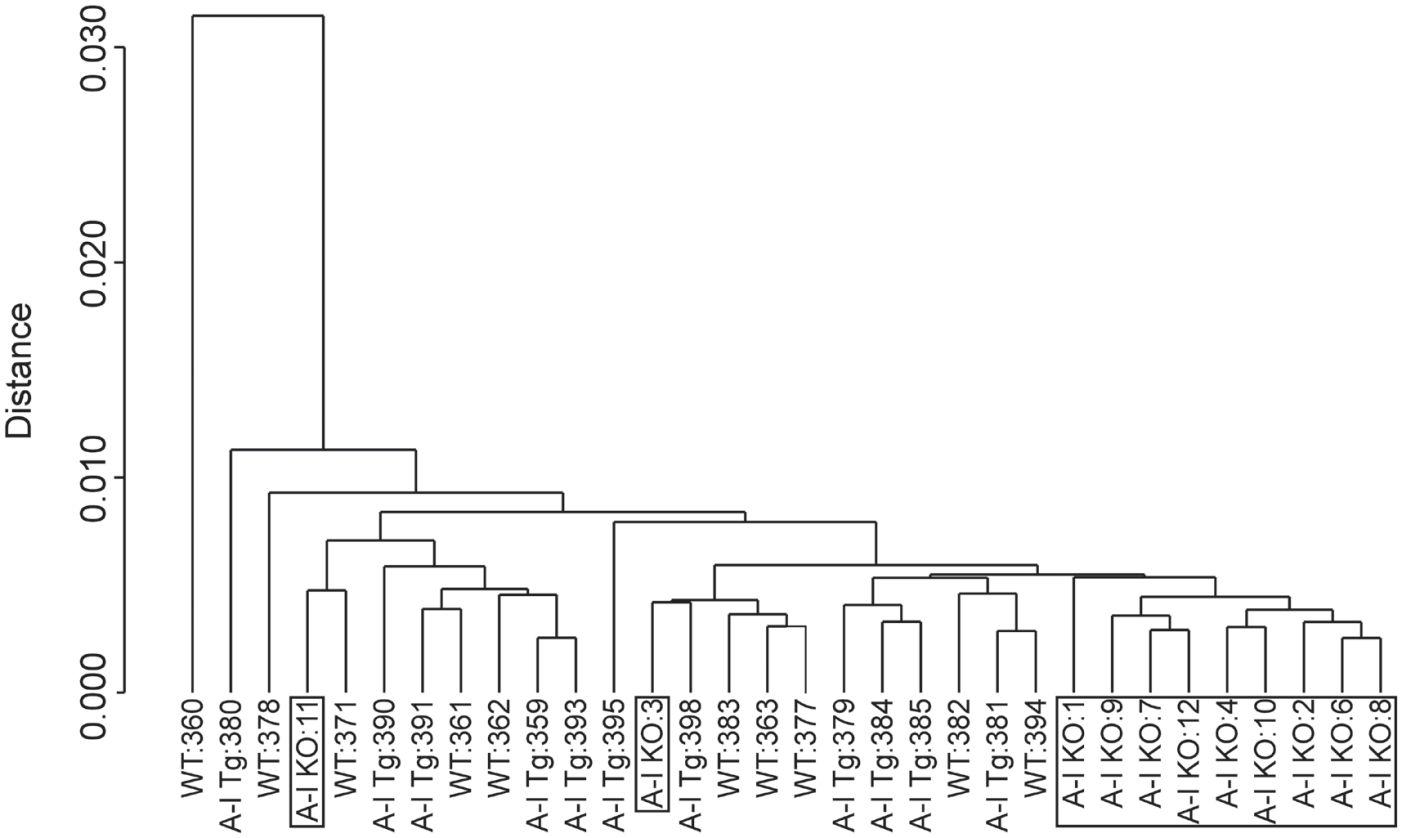
Dendogram of unsupervised hierarchical clustering analysis of tumor gene expression (log2 normalized) profiles discriminate A-I KO-derived tumors from less aggressive tumors of apoA-I-expressing mice (A-I Tg^+/–^ and WT). B16F10L melanoma homograft grown in C57BL/6 mice wild type for mouse apoA-I (WT), deficient (A-I KO) or expressing human apoA-I (A-I Tg^+/–^) were resected 7 days after inoculation and processed for RNA isolation and microarray analysis as described in Methods.

To gain insight into molecular mechanisms and biological pathways underlying apoA-I tumor suppressive activity, we performed whole-genome expression profiling of B16F10L homografts from A-I Tg^+/–^, A-I KO, and WT mice by DNA microarray technology (a total of 24,613 probes corresponding to 17,877 mouse genes were interrogated). Previously, we reported a significant difference in tumor-associated angiogenesis between A-I KO and A-I Tg^+/–^ seven days post tumor inoculation [16]. We therefore chose day 7 as a time point for transcriptomic analyses because adequate tumor mass could be harvested at this early time point. We compared 11 distinct primary tumors per genotype to compensate for variability when analyzing a heterogeneous population of cells, namely tumor and host tumor-infiltrating cells. The dendogram in Figure 1 illustrates the correlation analysis of log2 normalized gene expression levels where distance (y-axis) between clusters denotes divergence in the global gene expression pattern. While expression profiles of tumors from all three groups were generally similar, as reflected by the close distance between the clusters, for the most part the more aggressive A-I KO tumors clustered in a group distinct from the less aggressive tumors found in apoA-I-expressing animals (WT and A-I Tg^+/–^). Two of the eleven tumor gene signatures recovered from A-I KO animals, A-I KO 3 & 11, were exceptions in that they clustered within the A-I Tg^+/–^ and WT groups. One of the WT gene signatures recovered (WT 360) was an outlier in a cluster furthest in distance from all other tumors (Figure 1).

### Key biological pathways are dysregulated in B16F10L tumors from apoA-I null mice (A-I KO)

**Figure 2:**
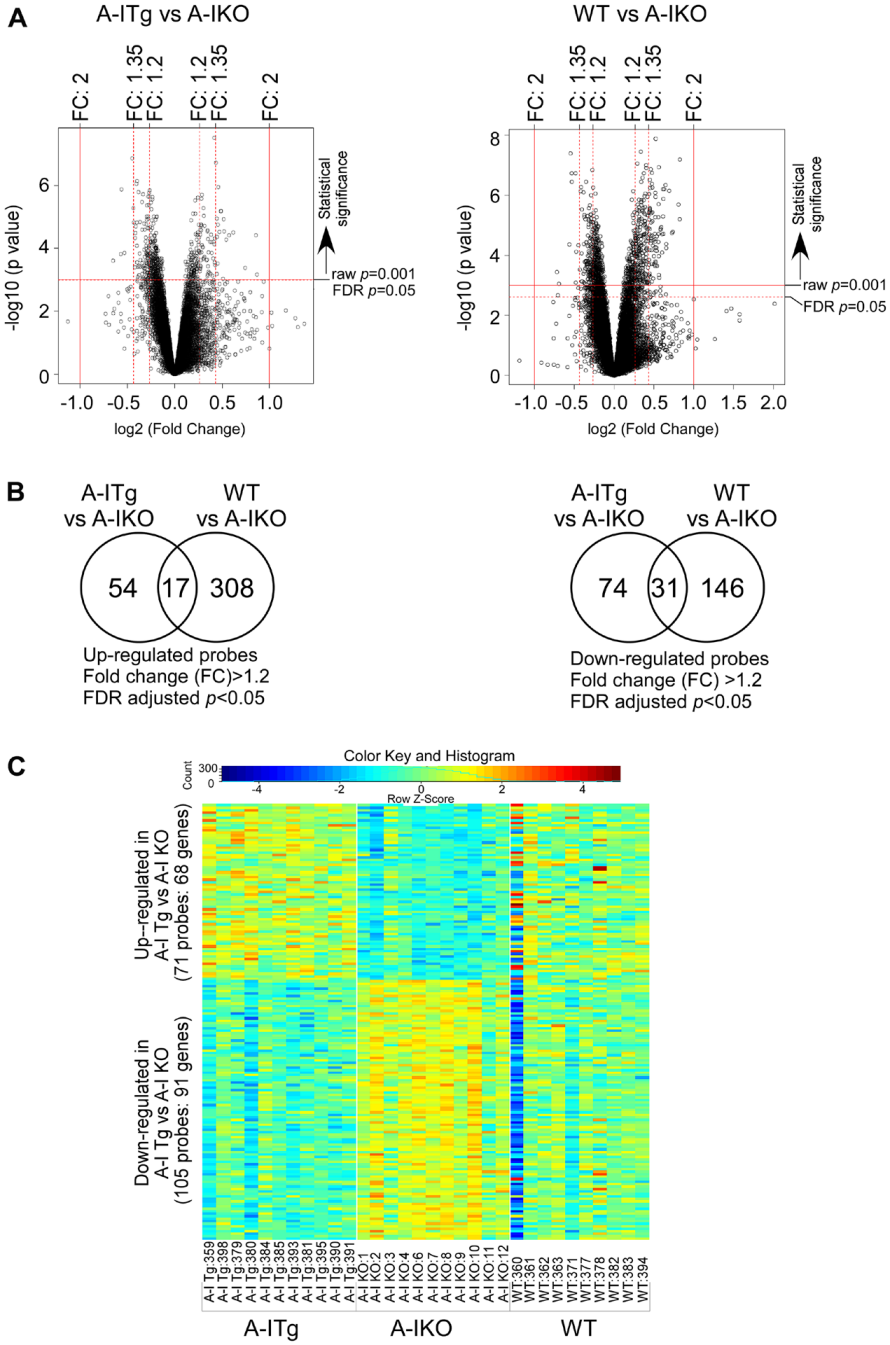
Identification of statistically significant transcripts from differential expression analyses. B16F10L melanoma homograft grown in C57BL/6 mice wild type for mouse apoA-I (WT), deficient (A-I KO) or expressing human apoA-I (A-I Tg^+/–^) were resected 7 days after inoculation and processed for RNA isolation and microarray analysis as described in Methods. (**A**) Volcano plots of expressed probes allowing for filtering of statistically significant (False Discover Rate (FDR) adjusted *p* < 0.05; Y-axis, -log10 (*p*-value)) and biologically significant probes (fold-change (FC), X-axis, log2(fold-change)). (**B**) Venn diagrams depicting the number of significant probes (FDR adjusted *p* < 0.05) that were up (left) or down (right) regulated (FC: 1.2) in A-I Tg^+/–^ or WT with respect to A-I KO. (**C**) Two-way hierarchical heat map of significant transcripts (FDR adjusted *p* < 0.05) with fold-change cut off > 1.2 resulting from comparison of B16F10L tumors from A-I Tg^+/–^ versus A-I KO mice. Columns refer to individual B16F10L primary tumors from host animals shown, and rows represent individual transcripts. Numbers following designated genotypes refer to unique mouse identifiers.

**Figure 3:**
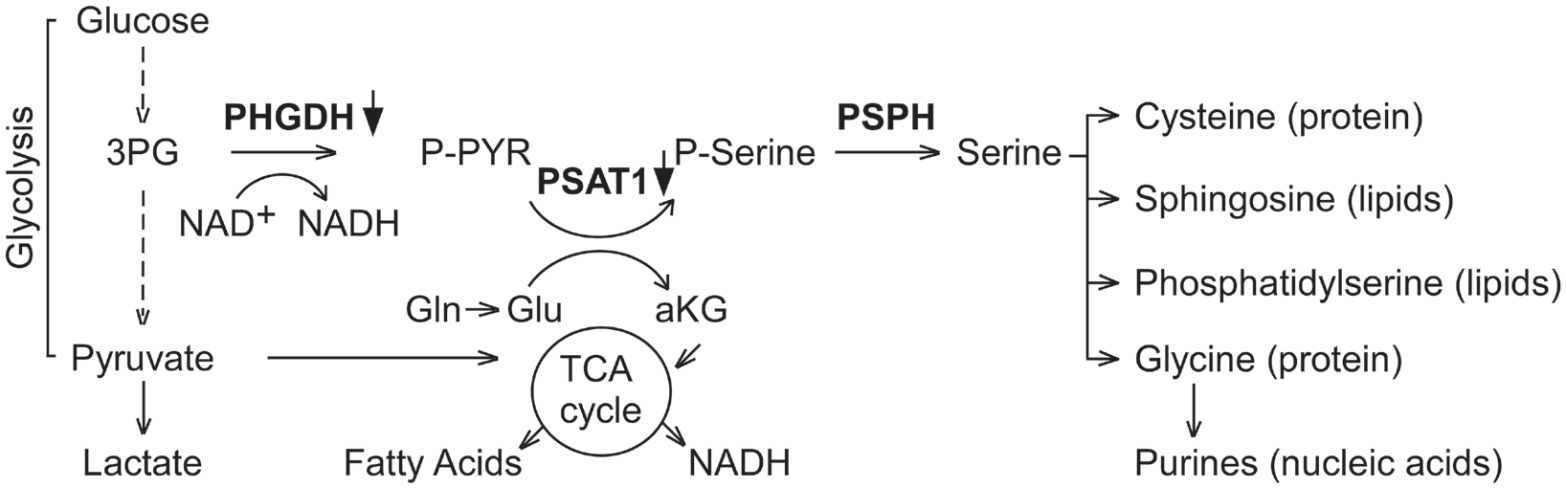
PHGDH, the first enzyme of *de novo* serine synthesis pathway, is the most repressed transcript in tumors from A-I Tg^+/–^ animals. Eleven separate primary tumors from eleven A-I Tg^+/–^ or A-I KO mice were subjected to differential gene expression analysis. Probes were considered significant if they met the criteria FDR adjusted *p* < 0.05 and fold-change (FC) cut off > 1.2. The complete list of significant genes is shown in Supplementary Tables 1 and 2. The transcripts for PHGDH and PSAT1 were down regulated (FDR adjusted *p*-value 0.027 and 0.104, respectively) by at least 1.2-fold in day 7 B16F10L tumor homograft from A-I Tg^+/–^ relative to A-I KO mice. 3PG: 3-Phosphoglycerate; PHGDH: 3-Phospho-glycerate dehydrogenase; P-PYR: 3-Phospho-hydroxypyruvate; Gln: Glutamine; Glu: Glutamate; aKG: a-Ketoglutarate; PSAT1: phosphoserine aminotransferase 1; P-Serine: Phosphoserine; PSPH: Phosphoserine phosphatase.

The expression array datasets between the tumor groups were analyzed (see Methods) to identify a cohort of genes that were differentially expressed. Volcano plots illustrating both statistical (y-axis, -log10 (*p*-value) and biological (x-axis, log2 (fold-change, FC)) significance of the differential expression analyses of A-I Tg^+/–^ versus A-I KO or WT versus A-I KO are shown in Figure 2A. We restricted our genes to those with at least 1.2-fold differential expression and a false discovery rate (FDR)-adjusted *p*-value < 0.05. Use of this screening criteria resulted in 71 and 325 upregulated, and 105 and 177 downregulated transcript probes comparing A-I Tg^+/–^ or WT versus A-I KO, respectively (Figure 2B). The heat map in Figure 2C illustrates the probes from differential expression analysis of A-I Tg^+/–^ versus A-I KO with the expression in WT, serving as reference, to the right. Importantly, the overall gene expression pattern in WT group was approximately mid-way between A-I Tg^+/–^ and A-I KO, supporting the tumor growth phenotype previously observed for this genotype relative to A-I KO and A-I Tg^+/–^ (Figure 1 in [16]). The transcripts representing 68 (71 probes) up- and 91 (105 probes) downregulated genes are listed in Supplementary Tables 1 and 2, respectively. Tables 1A and 1B represent the top genes up- or downregulated in tumors from A-I Tg^+/–^ versus A-I KO, respectively. Notably, *Phgdh,* the gene encoding phosphoglycerate dehydrogenase, PHGDH, was the most repressed transcript in tumors from apoA-I-expressing hosts (Table 1B). PHGDH is the first enzyme of the 3-step *de novo* serine synthesis pathway (Figure 3), which diverts glucose-derived carbon for synthesis of biomolecules necessary for rapid proliferation and survival. In this pathway, PHGDH oxidizes the glycolytic intermediate 3-phosphoglycerate (3PG) to 3-phospho-hydroxypyruvate (P-PYR) and couples to NADH production. Subsequently, P-PYR is transaminated by phosphoserine aminotransferase (PSAT1) with glutamate (Glu) serving as a nitrogen donor to form phosphoserine (P-Serine) and alpha-ketoglutarate (aKG), a key intermediate of the TCA cycle. P-Serine is dephosphorylated by phosphoserine phosphatase (PSPH) to form serine, which is essential for synthesis of glycine, proteins, lipids, and nucleic acids (Figure 3). Serine is also a major source of one-carbon units directed to the folate pool, which supports proliferation and transformation. The transcript for phosphoserine aminotransferase 1 (*Psat1*), the second enzyme of the *de novo* serine synthesis pathway, was similarly downregulated (Figure 3), however, the FDR-adjusted *p*-value (0.104) did not meet significance (*p* < 0.05).

**Table 1:** Top genes up- or downregulated in tumors from A-I Tg^+/–^ versus A-I KO

Table 1A: Upregulated genes in A-ITg vs A-IKO mice					
Probe_ID	Accession no.	Symbol	Name	FC	adj. *p*-value
ILMN_1251748	NM_139200.4	*Cytip*	cytohesin 1 interacting protein	1.83	0.04
ILMN_1253182	NM_010474.1	*Hs3st1*	heparan sulfate (glucosamine) 3-O-sulfotransferase 1	1.81	0.012
ILMN_2622983	NM_013642.2	*Dusp1*	dual specificity phosphatase 1	1.67	0.033
ILMN_3161601	NM_009221.2	*Snca*	synuclein, alpha (non A4 component of amyloid precursor)	1.54	0.011
ILMN_2705166	NM_145933.3	*St6gal1*	ST6 beta-galactosamide alpha-2,6-sialyltranferase 1	1.49	0.012
ILMN_2722732	NM_011157.2	*Srgn*	serglycin	1.46	0.015
ILMN_3136638	NM_009221.2	*Snca*	synuclein, alpha (non A4 component of amyloid precursor)	1.44	0.011
ILMN_1254031	NM_010638.4	*Klf9*	Kruppel-like factor 9	1.43	0.008
ILMN_3161105	NM_001033476.1	*Ahnak2*	AHNAK nucleoprotein 2	1.42	0.047
ILMN_2829594	NM_010479.2	*Hspa1a*	heat shock 70kDa protein 1A	1.4	0.006
ILMN_2754985	NM_009344.1	*Phlda1*	pleckstrin homology-like domain, family A, member 1	1.4	0.006
ILMN_2824971	NM_001004761.1	*Gpr158*	G protein-coupled receptor 158	1.39	0.011
ILMN_2896314	NM_015732.3	*Axin2*	axin 2	1.38	0.004
ILMN_1233064	NM_183417.2	*Cdk2*	cyclin-dependent kinase 2	1.37	0.009
ILMN_2892441	NM_010357.1	*Gsta4*	glutathione S-transferase alpha 4	1.37	0.029
ILMN_2831799	NM_019517.2	*Bace2*	beta-site APP-cleaving enzyme 2	1.36	0.013
ILMN_2623280	NM_011019.1	*Osmr*	oncostatin M receptor	1.36	0.008
ILMN_2522236	NM_011661.3	*Tyr*	tyrosinase	1.36	0.039
ILMN_3114585	NM_001039150.1	*Cd44*	CD44 molecule	1.35	0.002
ILMN_1252202	NM_009397.2	*Tnfaip3*	tumor necrosis factor, alpha-induced protein 3	1.34	0.03
ILMN_2612895	NM_013484.1	*C2*	complement component 2	1.34	0.014
ILMN_2680415	NM_172537.2	*Sema6d*	Semaphorin	1.34	0.028
ILMN_1246153	NM_133362.2	*Erdr1*	erythroid differentiation regulator 1	1.34	0.017
ILMN_1240323	NM_018808.1	*Dnajb1*	DnaJ (Hsp40) homolog, subfamily B, member 1	1.34	0.001
ILMN_2813484	NM_011065.2	*Per1*	period circadian clock 1	1.33	0.015
ILMN_2774690	XM_001004685.1	*LOC677317*	similar to NADP-dependent malic enzyme (NADP-ME) (Malic enzyme 1)	1.33	0.049
ILMN_2734181	NM_019811.3	*Acss2*	acyl-CoA synthetase short-chain family member 2	1.32	0.026
ILMN_1226157	NM_181585.5	*Pik3r3*	phosphoinositide-3-kinase, regulatory subunit 3 (gamma)	1.32	0.042
ILMN_2746556	NM_015814.2	*Dkk3*	dickkopf 3 homolog (Xenopus laevis)	1.31	0.022
ILMN_3073563	NM_001001884.1	*C230021P08Rik*	Nckap5l NCK-associated protein 5-like	1.31	0.015
ILMN_2776619	NM_008520.2	*Ltbp3*	latent transforming growth factor beta binding protein 3	1.3	0.012
ILMN_1258600	XM_001481024.1	*LOC100043671*	LOC100043671 hypothetical protein	1.3	0.04
ILMN_2870672	NM_010180.1	*Fbln1*	fibulin 1	1.3	0.015

Table 1B: Downregulated genes in A-ITg vs A-IKO mice					
Probe_ID	Accession no.	Symbol	Name	FC	adj. *p*-value
ILMN_2790181	NM_016966.3	*Phgdh*	phosphoglycerate dehydrogenase	1.57	0.027
ILMN_3081854	NM_001025245.1	*Mbp*	myelin basic protein	1.48	0.023
ILMN_2600348	NM_009270.3	*Sqle*	squalene epoxidase	1.47	0.004
ILMN_2634905	NM_007994.3	*Fbp2*	fructose-1,6-bisphosphatase 2	1.45	0.022
ILMN_2772274	NM_001079694.1	*Sfrs5*	serine/arginine-rich splicing factor 5	1.37	0.002
ILMN_2688075	NM_020010.2	*Cyp51*	cytochrome P450, family 51	1.35	0.005
ILMN_3148662	NM_001079695.1	*Sfrs5*	serine/arginine-rich splicing factor 5	1.35	0.009
ILMN_2712557	NM_024439.3	*H47*	histocompatibility 47 Gene	1.34	0.032
ILMN_2996877	NM_010063.1	*Dync1i1*	dynein cytoplasmic 1 intermediate chain 1	1.34	0.013
ILMN_2700292	NM_010376.3	*H13*	histocompatibility 13	1.33	0.011
ILMN_2630641	NM_009272.4	*Srm*	spermidine synthase	1.33	0.009
ILMN_2737163	NM_009270.3	*Sqle*	squalene epoxidase	1.32	0.004
ILMN_2761594	NM_007705.2	*Cirbp*	cold inducible RNA binding protein	1.32	0.009
ILMN_2660414	NM_025442.3	*Alg5*	ALG5, dolichyl-phosphate beta-glucosyltransferase	1.32	0.018
ILMN_3086899	NM_198104.2	*Tcte3*	t-complex-associated-testis-expressed 3	1.32	0.004
ILMN_2823778	NM_025436.1	*Sc4mol*	(testis meiosis-activating sterol/sterol C4-methyl oxidase–like)	1.32	0.028
ILMN_2642417	NM_008590.1	*Mest*	mesoderm specific transcript	1.31	0.048
ILMN_1247916	NM_144862.3	*Lims2*	LIM and senescent cell antigen-like domains 2	1.31	0.044
ILMN_2739825	NM_010063.1	*Dync1i1*	dynein cytoplasmic 1 intermediate chain 1	1.31	0.032
ILMN_1229529	NM_010476.3	*Hsd17b7*	hydroxysteroid (17-beta) dehydrogenase 7	1.3	0.004
ILMN_2606693	NM_153526.4	*Insig1*	insulin induced gene 1	1.3	0.031
ILMN_1238654	NM_027352.3	*Gorasp2*	golgi reassembly stacking protein 2	1.3	0.041

(A) Upregulated genes. (B) Downregulated genes. Eleven separate primary tumors from eleven A-I Tg^+/–^ or A-I KO mice were subjected to differential gene expression analysis. Probes were considered significant if they met the criteria FDR adjusted *p* < 0.05 and fold-change (FC) cut off > 1.2. The complete list of significant genes is shown in Supplementary Tables 1 and 2.

**Figure 4:**
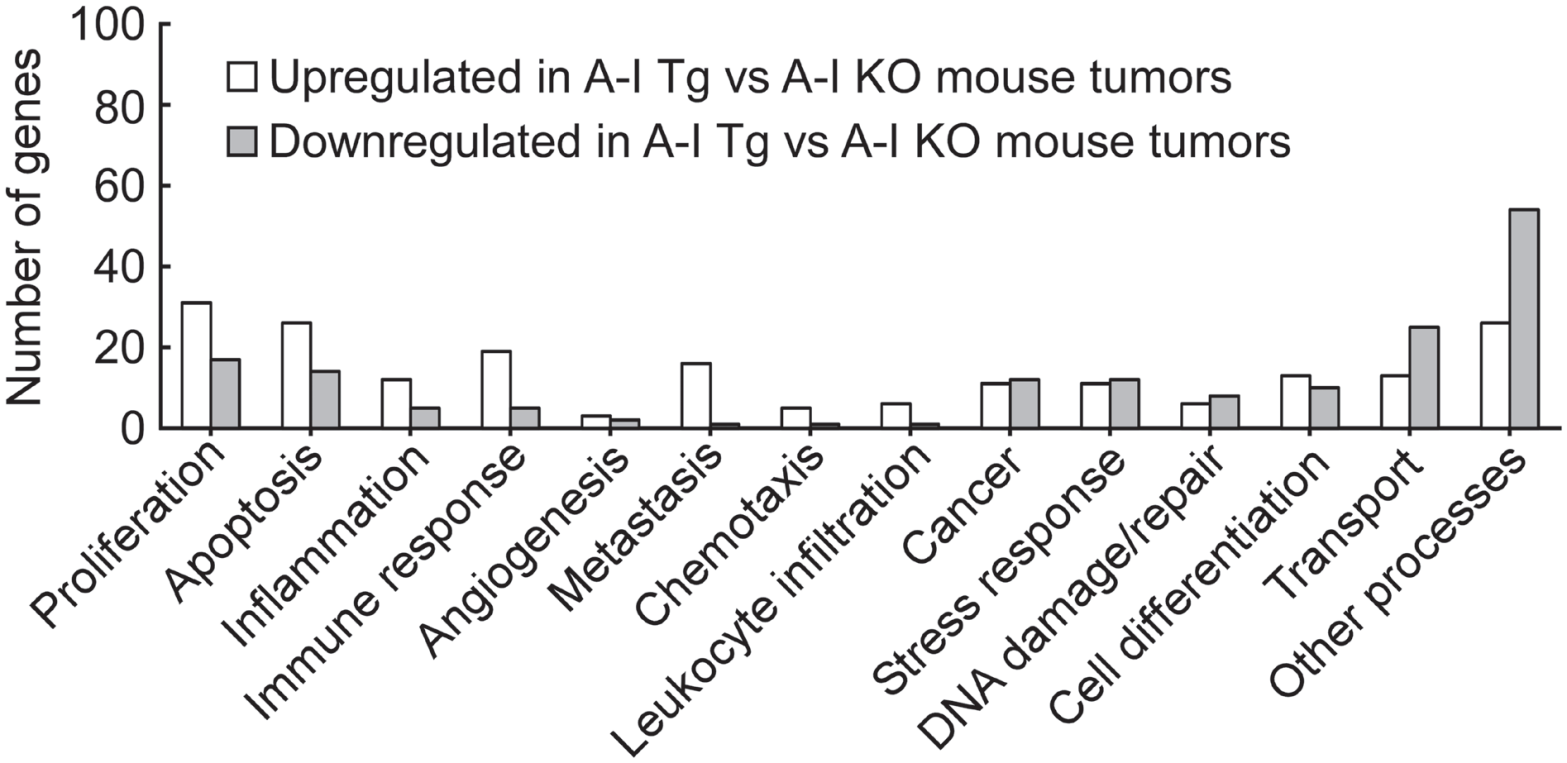
Genes from differential analysis of B16F10L tumors from A-I Tg^+/–^ versus A-I KO mapped to key cancer related biological processes. The significant genes from differential analysis of day 7 B16F10L tumors from A-I Tg^+/–^ versus A-I KO mice (listed in Supplementary Tables 1 and 2) were subjected to bioinformatics analysis. The mapping of genes to key cancer-related processes was obtained from disparate functional annotation data sources mentioned in the Methods section.

To gain further insight into molecular mechanisms that might impart a growth advantage to tumors in the absence of host apoA-I, we compared the differentially-regulated genes listed in Supplementary Tables 1 and 2 to publically available, as well as proprietary annotated, melanoma databases for functional analyses (see Methods). Initially, we queried our list in a supervised fashion to an assortment of key biological processes relevant to cancer. These are shown in Figure 4, and the genes on this list that associated with these processes are listed in Table 2. Some genes, for example *Dicer*, *Adamts1*, and *Cdc20*, which were all downregulated in A-I Tg^+/–^, mapped to more than one biological process.

**Table 2:** Genes from differential analysis of B16F10L tumors from A-I Tg^+/–^ versus A-I KO mapped to key cancer related biological processes

Biological Processes	Up-regulated genes in A-ITg	Down-regulated genes in A-ITg
Proliferation	*Axin2, Cd44, Cd80, Cdk2, Cxcr4, Dnajb1, Dusp1, Enpp2, Fbln1, Hspa1a, Irx3, Irx5, Klf9, Ltbp3, Mapt, Mitf, Mknk2, Nfat5, Osmr, Peli1, Per1, Phlda1, Pik3r3, Ptprs, Sort1, Srebf1, Srgn, St6gal1, Tnfaip3, Tob2, Tyr*	*Adamts1, Apex1, Cdc20, Cirbp, Dhcr24, Dicer1, Eif5a, Exosc4, Fadd, Gorasp2, Mbp, Mcm3, Mvd, Phgdh, Slc19a1, Tmpo, Vti1a*
Apoptosis	*Axin2, Cd44, Cdk2, Cxcr4, Dnajb1, Dusp1, Enpp2, Fbln1, Hspa1a, Ltbp3, Luc7l3, Map3k12, Mapt, Mitf, Nfat5, Peli1, Per1, Phlda1, Pik3r3, Ptprs, Snca, Sort1, Srgn, St6gal1, Tnfaip3, Tyr*	*Alg5, Apex1, Cdc20, Dhcr24, Dhx9, Dicer1, Eif5a, Fadd, H47, Lims2, Mbp, Slc19a1, Tcte3, Ubqln1*
Inflammation	*C2, Cd44, Cdk2, Cxcr4, Dusp1, Osmr, Per1, Srebf1, Srgn, St6gal1, Tnfaip3, Tyr*	*Dicer1, Fadd, H47, Mbp, Stx18*
Immune response	*C2, Cd44, Cd80, Cdk2, Cxcr4, Dusp1, Enpp2, Hspa1a, Klf9, Mitf, Osmr, Peli1, Per1, Snca, Srebf1, St6gal1, Tnfaip3, Tob2, Tyr*	*Dicer1, Fadd, H47, Mbp, Slc19a1*
Angiogenesis	*Robo4, Cd44, Cxcr4*	*Dicer1, Wars*
Metastasis	*C2, Cd44, Cxcr4, Dusp1, Enpp2, Fbln1, Map3k12, Mapt, Nfat5, Pik3r3, Robo4, Sema6d, Sort1, Srebf1, St6gal1, Tnfaip3*	*Apex1*
Chemotaxis	*Cd44, Cytip, Enpp2, Ptprs, Dusp1*	*Pcdh17*
Leukocyte infiltration	*Cd44, Cxcr4, Cytip, Mitf, Pik3r3, Snca*	*Dicer1*
Cancer	*Cd44, Cdk2, Cxcr4, Enpp2, Klf9, Mitf, Osmr, Pik3r3, Srgn, Tnfaip3, Tyr*	*Adamts1, Cdc20, Cirbp, Dicer1, Fadd, Insig1, Mcm3, Mvd, Nme4, Slc19a1, Sqle, Srm*
Stress response	*Axin2, C2, Cd44, Dnajb1, Hspa1a, Insig2, Luc7l3, Peli1, Snca, Srebf1, Tnfaip3*	*Apex1, Cirbp, Dhx9, Dicer1, Fadd, H47, Insig1, Mpv17, Rad51ap1, Sfrs5, Ubqln1, Wfdc12*
DNA damage/repair	*Cdk2, Dnajb1, Dusp1, Hspa1a, Map3k12, Srgn*	*Apex1, Cdc20, Dicer1, Mcm3, Nme4, Nup107, Slc19a1, Tmpo*
Cell differentiation	*Axin2, Cd44, Cxcr4, Erdr1, Irx3, Irx5, Ltbp3, Mapt, Mitf, Robo4, Sema6d, Sort1, Tob2*	*AI428936, Camk1, Cdc20, Dicer1, Eif5a, Insig1, Mbp, Nle1, Phgdh, Stk25*
Transport	*Acsl3, Axin2, Cd44, Cdk2, Cxcr4, Elovl5, Insig2, Mapt, Ptprs, Snca, Sort1, Srebf1, Srgn*	*Apex1, Camk1, Dhcr24, Dync1i1, Eif5a, Fadd, H47, Il1rl1l, Insig1, Mbp, Mybbp1a, Nup107, Phgdh, Preb, Rhobtb3, Slc19a1, Snf8, Srpr, Stard4, Stard5, Stx18, Tmed4, Vti1a, Yif1a, Zfpl1*
Other Processes	*2700097009Rik, Acss2, Ahnak2, Bace2, Clmn, Crem, Dkk3, Efcab6, Eif4a2, Fbxo30, Gcnt2, Gpr158, Gsta4, Hs3st1, Lekr1, Loc100043671, Loc100046802, Loc677317, Nckap5l, Plekha3, Rdm1, Tex2, Ube2o, Unc119b, Zfp597, Zmynd8*	*Get4, 1810065E05Rik, 2410002F23Rik, Fam176b, 3010003L21Rik, Trmt61a, Bud31, C85627, Cyp51a1, Dus1l, Elovl1, Fbp2, Fbxo36, Gale, Gmpr, Grwd1, Hm13, Hsd17b7, Oc100040592, Loc100043257, Mest, Mpdu1, Mrps18b, Msto1, Nif3l1, Nol12, Nop56, Mak16, Rnf144a, Rnf25, Rwdd3, Sc4mol, Smyd5, Snrpa, Sssca1, Sumo3, Tmem17, Tmem214, Ttc39a, Znf32*

List of genes mapped to key cancer-related processes in Figure 4. List of significant genes mapped to processes relevant to cancer.

**Figure 5:**
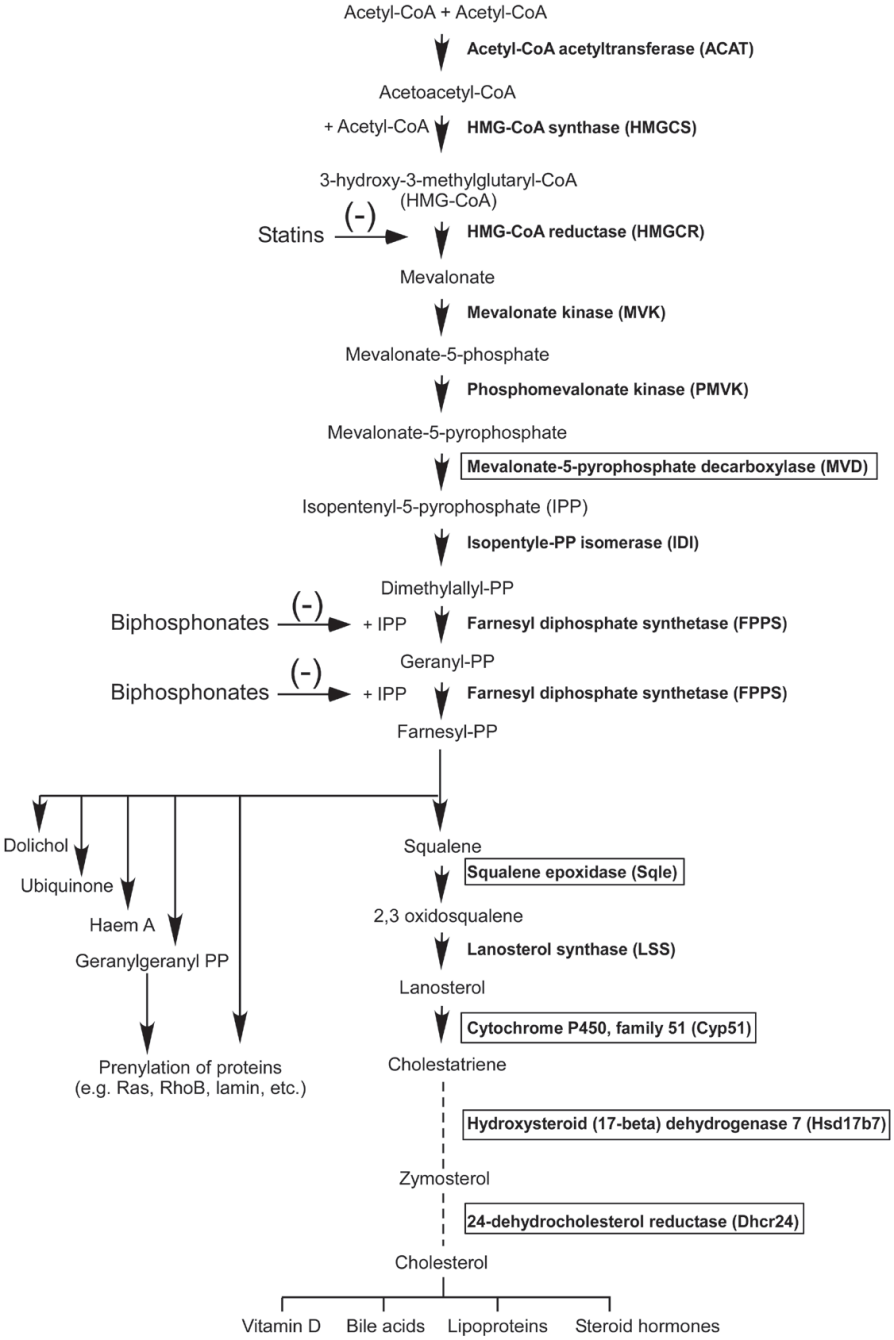
The mevalonate pathway. The transcripts for enzymes in boxes were down regulated (FDR adjusted *p* < 0.05) by at least 1.2-fold in day 7 melanoma tumor homograft from A-I Tg^+/–^ relative to A-I KO mice.

The Ingenuity Pathway Analysis (IPA) program was then used to generate biological networks from functional relationships between genes or proteins based on the published literature (http://www.Ingenuity.com). It identified lipid and cholesterol synthesis as top relevant pathways for genes downregulated in tumors from A-I Tg^+/–^ mice (Supplementary Figure 1A, 1B and Supplementary Table 3A and 3B). Significantly, five of these genes (*Mvd, Sqle, Cyp51, Hsd17b7,* and *Dhcr24)* are within the core biochemical processes of the mevalonate pathway, an important target for anti-cancer therapy [24, 27]. The mevalonate pathway converts acetyl-CoA via mevalonate, the product of the tightly regulated enzyme HMG-CoA reductase and the target of statins, into a variety of end products fundamental to cellular and organismal life (Figure 5).

**Figure 6:**
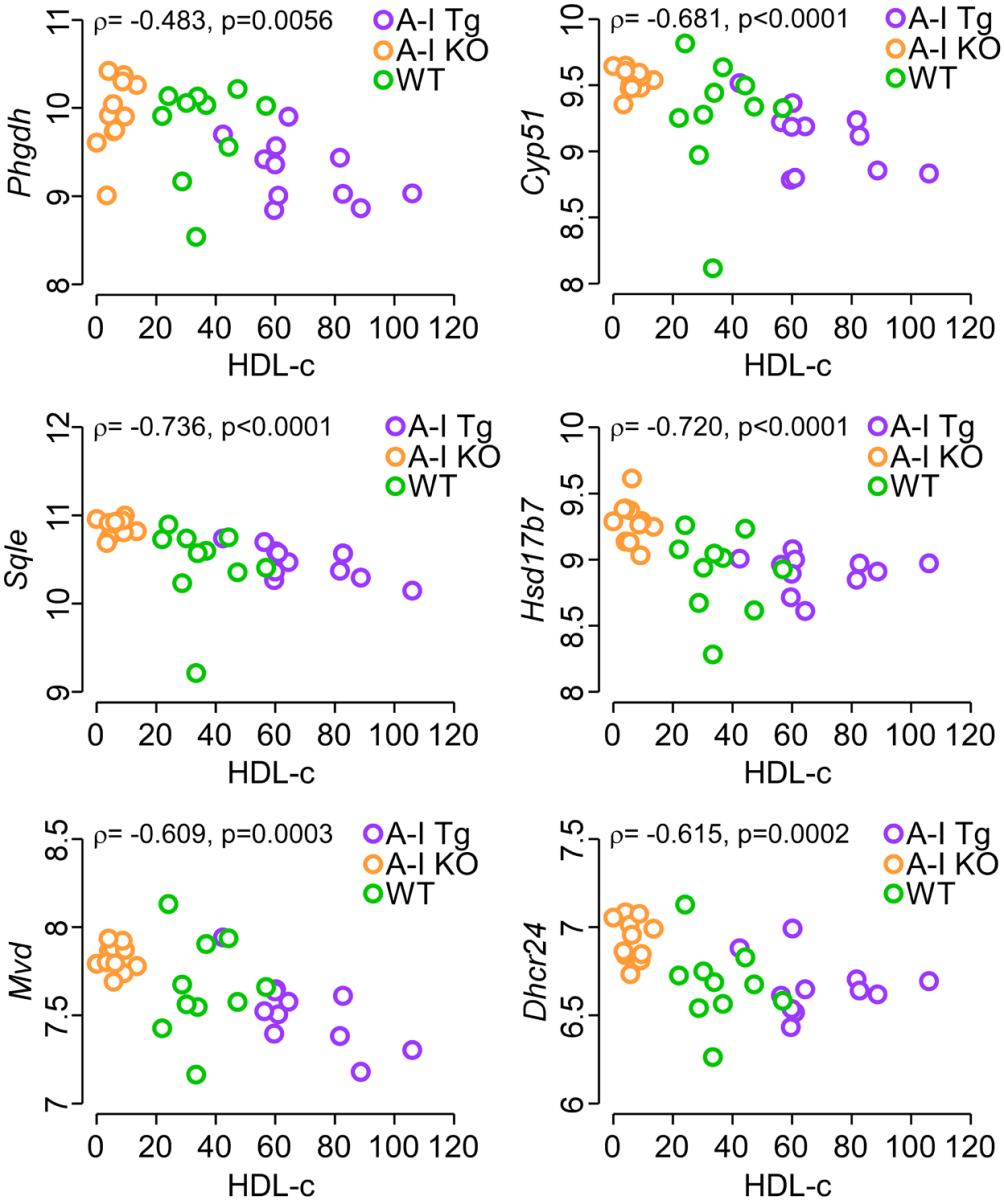
Inverse correlation between circulating HDL-c levels in tumor-bearing mice and tumor transcript levels of *Phgdh*, the first enzyme of serine synthesis pathway as well as enzymes of the mevalonate pathway. The gene chip expression signal of the indicated genes after positive background correction, log2 transformation, and quantile normalization was plotted against HDL-c levels (see Methods) in tumor bearing mice. Each data point represents the transcript signal from one tumor-bearing animal. The *p*-value were calculated with Spearman’s rank correlation and analyses were performed using R 3.5.3.

We next tested whether or not a dose-dependent expression correlation existed between each of *Phgdh*, *Mvd, Sqle, Cyp51, Hsd17b7, Dhcr24* and HDL-c levels. Figure 6 shows a significant inverse association between transcript levels of all six genes and HDL-c consistent with the notion that higher circulating apoA-I (HDL-c) levels inhibit expression of these genes in the developing tumor.

### Gene expression profiles in tumors from A-I KO are concordant with human melanoma gene signature for adverse outcome

We previously reported that apoA-I therapy was effective against human melanoma in nude mice [16]. We next compared the gene expression data mined in this study with published human melanoma gene signatures [8] to determine whether genes known to track with poor prognosis in humans are concordant with those found in tumors in the A-I KO mice, the more aggressive tumor setting in our mouse model. Human microarray studies [8] identified the expression profile of genes associated with 4-year distant metastasis-free survival, metastasis, or death among 58 patients with 4-yr follow up. Of the 250 distinct genes identified by this human study, a total of 170 were eligible for comparison in our mouse study; the remaining 80 genes were not present on the mouse array. Of the 170 genes under consideration, 40 did not give any detectable signal on our array, leaving 130 genes for comparison with the expression dataset in this study. We interrogated the eligible genes in our day 7 gene expression dataset with respect to fold-change (A-I KO/A-I Tg^+/–^) in expression signal normalized to beta-2-microglobulin with a *p*-value cut off < 0.05 (Student’s *t*-test). Remarkably, 45% (58) of human genes reported to associate with poor prognosis were also concordant in tumors from A-I KO animals with only 3% (4 genes) going in the opposite direction. We observed no significant difference between A-I KO and A-I Tg^+/–^ in the remaining 52% (68 genes). Table 3 lists the genes compared in the human and mouse study and their expression trend. Table 4 maps the genes to top functions in IPA: cell cycle, DNA replication and repair, nucleic acid metabolism, and cellular assembly and organization as well as cancer. The genes listed in Table 3 were also observed to be differentially regulated in two independent datasets, Riker and Talantov studies [7, 40] from the Oncomine datasets of cutaneous melanoma versus normal (Table 5). Forty-one (41) out of 58 concordant genes in Table 3 were observed to be differentially regulated (> 2-fold change) and statistically significant (*p* < 0.01) in these independent datasets. Of the genes upregulated in A-I KO (40 out of 41), 12 were concordant in both Riker and Talantov studies [7, 40] and 27 were concordant in either Riker or Talantov [7, 40] (Table 5). Only one gene, the nuclear hormone receptor-interacting protein nuclear receptor coactivator 6 (*Ncoa6*), was non-concordant, being upregulated in our dataset but downregulated in the Talantov study (Table 5). Gene *F10*, which encodes the vitamin K-dependent coagulation factor X of the blood coagulation pathway, was downregulated in our study and was also repressed in the Talantov data set (Table 5), as were the human melanoma signatures [8]. These comparisons with three distinct human melanoma studies, as well as the observed high degree of concordance of genes both upregulated or downregulated in melanomas associated with adverse outcomes with published human melanoma gene signatures, validate the mouse gene expression dataset results presented here.

**Table 3:** A significant number of genes associated with poor prognosis in human melanoma were also concordant in B16F10L tumors from A-I KO mice

PROBE_ID	SYMBOL	DEFINITION	FOLD CHANGE A-IKO/A-ITg	*p*-value	Expression associated with adverse prognosis in humans
ILMN_1231392	*Melk*	maternal embryonic leucine zipper kinase (Melk), mRNA.	1.5	0.002	Up regulated
ILMN_2804444	*Pcdh17*	protocadherin 17 (Pcdh17), mRNA.	1.5	0.001	Up regulated
ILMN_2741050	*Rfc4*	replication factor C (activator 1) 4 (Rfc4), mRNA.	1.4	0.013	Up regulated
ILMN_2757224	*Srp19*	signal recognition particle 19 (Srp19), mRNA.	1.4	0.005	Up regulated
ILMN_2711112	*Shcbp1*	Shc SH2-domain binding protein 1 (Shcbp1), mRNA.	1.4	0.001	Up regulated
ILMN_2878355	*Kpna2*	karyopherin (importin) alpha 2 (Kpna2), mRNA.	1.4	0.005	Up regulated
ILMN_2607926	*Kdelr2*	KDEL (Lys-Asp-Glu-Leu) endoplasmic reticulum protein retention receptor 2 (Kdelr2), mRNA.	1.4	0.002	Up regulated
ILMN_2714565	*Rrm2*	ribonucleotide reductase M2 polypeptide	1.4	0.002	Up regulated
ILMN_2756008	*Nasp*	nuclear autoantigenic sperm protein (histone-binding)	1.4	0.002	Up regulated
ILMN_2816754	*Ndc80/Kntc2*	NDC80 homolog, kinetochore complex component (S. cerevisiae) (Ndc80), mRNA.	1.4	0.006	Up regulated
ILMN_2971845	*Dtymk*	deoxythymidylate kinase (Dtymk), mRNA.	1.4	0.006	Up regulated
ILMN_2588362	*Cdca8*	cell division cycle associated 8 (Cdca8), mRNA.	1.4	0.003	Up regulated
ILMN_2934457	*Ran*	RAN, member RAS oncogene family (Ran), mRNA.	1.4	0.004	Up regulated
ILMN_2688944	*Htra2/Prss25*	HtrA serine peptidase 2 (Htra2), nuclear gene encoding mitochondrial protein, mRNA.	1.4	0.001	Up regulated
ILMN_1214319	*Gemin6*	gem (nuclear organelle) associated protein 6 (Gemin6), mRNA.	1.4	0.001	Up regulated
ILMN_2632712	*Birc5*	baculoviral IAP repeat-containing 5 (Birc5), transcript variant 1, mRNA.	1.4	0.006	Up regulated
ILMN_2999654	*Psmc3ip*	proteasome (prosome, macropain) 26S subunit, ATPase 3, interacting protein (Psmc3ip), mRNA.	1.4	0.003	Up regulated
ILMN_2780177	*Mrps5*	mitochondrial ribosomal protein S5 (Mrps5), nuclear gene encoding mitochondrial protein, mRNA.	1.4	0.005	Up regulated
ILMN_1244296	*Cdc14b/Znf367*	CDC14 cell division cycle 14 homolog B (S. cerevisiae) (Cdc14b), mRNA.	1.4	0.024	Up regulated
ILMN_1257552	*Tars*	threonyl-tRNA synthetase (Tars), mRNA.	1.4	0.008	Up regulated
ILMN_2621422	*Kirrel*	kin of IRRE like (Drosophila) (Kirrel), mRNA.	1.3	0.026	Up regulated
ILMN_2982965	*Prim2*	DNA primase, p58 subunit (Prim2), mRNA.	1.3	0.005	Up regulated
ILMN_2598852	*Ranbp1*	RAN binding protein 1 (Ranbp1), mRNA.	1.3	0.007	Up regulated
ILMN_3097131	*Timeless*	timeless homolog (Drosophila) (Timeless), transcript variant 2, mRNA.	1.3	0.013	Up regulated
ILMN_2595846	*Surf4*	surfeit gene 4 (Surf4), mRNA.	1.3	0.003	Up regulated
ILMN_2639036	*Hspd1*	heat shock protein 1 (chaperonin) (Hspd1), mRNA.	1.3	0.011	Up regulated
ILMN_2745005	*Gpn3/MGC14560*	GPN-loop GTPase 3 (Gpn3), mRNA.	1.3	0.005	Up regulated
ILMN_2619671	*Mrps16*	mitochondrial ribosomal protein S16 (Mrps16), nuclear gene encoding mitochondrial protein, mRNA.	1.3	0.011	Up regulated
ILMN_2787871	*Cdca5*	cell division cycle associated 5 (Cdca5), mRNA.	1.3	0.008	Up regulated
ILMN_2768984	*Anln*	anillin, actin binding protein (Anln), mRNA.	1.3	0.032	Up regulated
ILMN_2484707	*Tyms*	thymidylate synthase (Tyms), mRNA.	1.3	0.013	Up regulated
ILMN_2919433	*Cdc45l*	cell division cycle 45 homolog (S. cerevisiae)-like (Cdc45l), mRNA.	1.3	0.043	Up regulated
ILMN_1218967	*Kif2c*	kinesin family member 2C (Kif2c), mRNA.	1.3	0.015	Up regulated
ILMN_1233857	*Mcm6*	minichromosome maintenance deficient 6 (MIS5 homolog, S. pombe) (S. cerevisiae) (Mcm6), mRNA.	1.3	0.012	Up regulated
ILMN_2749937	*Ncoa6*	nuclear receptor coactivator 6 (Ncoa6), mRNA.	1.3	0.011	Up regulated
ILMN_2797642	*Ncaph/Brrn1*	non-SMC condensin I complex, subunit H (Ncaph), mRNA.	1.3	0.013	Up regulated
ILMN_2637203	*Rfc5*	replication factor C (activator 1) 5 (Rfc5), mRNA.	1.3	0.022	Up regulated
ILMN_2758690	*Mrps17*	mitochondrial ribosomal protein S17 (Mrps17), mRNA.	1.3	0.012	Up regulated
ILMN_2706882	*Donson*	downstream neighbor of SON (Donson), mRNA.	1.3	0.028	Up regulated
ILMN_2698282	*Fusip1*	FUSiinteracting protein	1.3	0.010	Up regulated
ILMN_2680648	*Atad2*	ATPase family, AAA domain containing 2 (Atad2), mRNA.	1.3	0.026	Up regulated
ILMN_2983686	*Trub2*	TruB pseudouridine (psi) synthase homolog 2 (E. coli) (Trub2), mRNA.	1.3	0.017	Up regulated
ILMN_2657844	*Cdc2a*	cell division cycle 2 homolog A (S. pombe) (Cdc2a), mRNA.	1.3	0.021	Up regulated
ILMN_2605890	*Tk1*	thymidine kinase 1 (Tk1), mRNA.	1.3	0.036	Up regulated
ILMN_2677595	*Ncapg2/Mtb*	non-SMC condensin II complex, subunit G2 (Ncapg2), mRNA.	1.2	0.022	Up regulated
ILMN_2608933	*Tcof1*	Treacher Collins Franceschetti syndrome 1, homolog (Tcof1), mRNA.	1.2	0.030	Up regulated
ILMN_1233065	*Rbmx*	RNA binding motif protein, X chromosome (Rbmx), mRNA.	1.2	0.053	Up regulated
ILMN_3163044	*Ola1/PTD004*	Obg-like ATPase 1 (Ola1), transcript variant 1, mRNA.	1.2	0.016	Up regulated
ILMN_1236574	*Cenpa*	centromere protein A (Cenpa), mRNA.	1.2	0.016	Up regulated
ILMN_3162184	*AU014645/Ncbp1*	expressed sequence AU014645 (AU014645), mRNA.	1.2	0.010	Up regulated
ILMN_1221067	*Nme1*	non-metastatic cells 1, protein (NM23A) expressed in (Nme1), mRNA.	1.2	0.006	Up regulated
ILMN_2830661	*Top2a*	topoisomerase (DNA) II alpha (Top2a), mRNA.	1.2	0.025	Up regulated
ILMN_2975640	*Snrpg*	small nuclear ribonucleoprotein polypeptide G (Snrpg), mRNA.	1.2	0.013	Up regulated
ILMN_1245757	*Nans*	N-acetylneuraminic acid synthase (sialic acid synthase) (Nans), mRNA.	1.2	0.027	Up regulated
ILMN_2717172	*Dph3/Zcsl2*	DPH3 homolog (KTI11, S. cerevisiae) (Dph3), transcript variant 2, mRNA.	1.2	0.043	Up regulated
ILMN_2936427	*Mcm4*	minichromosome maintenance deficient 4 homolog (S. cerevisiae) (Mcm4), mRNA.	1.2	0.050	Up regulated
ILMN_1242622	*Crem*	cAMP responsive element modulator (Crem), mRNA.	0.7	0.002	Up regulated
ILMN_2631423	*H2-Ab1/HLA-DQB1*	histocompatibility 2, class II antigen A, beta 1 (H2-Ab1), mRNA.	0.6	0.005	Down regulated
ILMN_2688912	*F10*	coagulation factor X	0.4	0.012	Down regulated
ILMN_1258462	*Hoxa9*	homeo box A9 (Hoxa9), mRNA.	1.5	0.031	Down regulated
ILMN_2655373	*Siat7f*	CMP-NeuAC:(b)-N-acetylgalactosaminide (a)2,6-sialyltransferase member VI	1.5	0.004	Down regulated
ILMN_1232182	*Ctnnbip1*	catenin beta interacting protein 1 (Ctnnbip1), mRNA.	1.3	0.020	Down regulated

One-hundred-thirty (130) human genes previously identified as part of a gene signature associated with poor prognosis in melanoma [8] were compared with genes identified in this study from the differential analysis of day 7 B16F10L melanoma tumors with respect to fold-change (A-I KO/A-I Tg^+/–^) in expression signal normalized to beta-2-microglobulin with a *p*-value cut off < 0.05 (Student’s *t*-test). The human microarray study identified the expression profile of genes associated with 4-year distant metastasis-free survival, metastasis, or death among 58 patients with 4-yr follow up [8]. Of the 250 human genes identified only 130 were eligible for comparison in our current study. Here we provide a list of genes in our study that were compared with the human microarray study. Boxes denote genes that were not concordant in the two studies. Genes with significant difference in expression between A-IKO and A-ITg.

**Table 4:** Mouse genes differentially regulated in B16F10L tumors from A-I KO and A-I Tg^+/–^ mice and concordant with human genes associated with poor outcome in melanoma were mapped to top functions in IPA

Expression in A-I KO relative to A-I Tg mice	Gene Symbol	Top Functions
**↑**	*Atad2, Birc5, Cdca8, Cdk1, Cenpa, Dtymk, Htra2, Kif2c, Mcmr, Mcm6, Melk, Nasp, Ndc80, Psmc3ip, Ran, Ranbp1, Rrm2, Srp19, Timeless, Tyms*	DNA Replication, Recombination and Repair, Cellular Assembly and Organization, Nucleic Acid Metabolism
**↓**	*F10, Hla-dqb1*	
**↑**	*Anln, Atad2, Cdc45, Cdc14b, Gpn3, Kpna2, Mrps16, Mrps17, Nans, Ncapg2, Ncaph, Rfc4, Shcbp1, Tyms*	Cell Cycle, DNA Replication, Recombination and Repair, Cellular Assembly and Organization
**↑**	*Kdelr2, Loc100505793/Fusip1, Ncp1, Ncoa6, Ola1, Prim2, Rbmx, Rfc5, Srp19, Surf4, Tars, Trub2*	Cell Cycle, Cancer, Infection Mechanism
**↑**	*Cdca5, Gemin6, Hspd1, Nme1, Rfc4, Rfc5, Snrpg, Tcof1, Tk1, Top2a*	Cell Cycle, DNA Replication, Recombination and Repair, Developmental Disorder

Genes whose expression in A-I KO relative to A-I Tg^+/–^ were determined to be concordant with a subset of genes previously identified in a human study [8] to track with poor outcome in melanoma (Table 3), were mapped to top functions in Ingenuity Pathway Analysis (IPA) and are shown in Table 4. Arrows indicate the direction of expression of these genes in day 7 B16F10L tumors from A-I KO relative to A-I Tg^+/–^ animals.

**Table 5:** Gene expression profiles in tumors from A-I KO are concordant with human melanoma gene signature for adverse outcome

Probe_ID	Gene Symbol	DEFINITION	A-IKO/ A-ITg (Mean)	*p*-value	Expression (in this study and in Oncomine’s)	Oncomine Datasets- Cutaneous Melanoma vs normal
ILMN_1231392	*Melk*	Mus musculus maternal embryonic leucine zipper kinase (Melk), mRNA.	1.5	0.002	↑↑	Riker, Talantov
ILMN_2878355	*Kpna2*	Mus musculus karyopherin (importin) alpha 2 (Kpna2), mRNA.	1.4	0.005	↑↑	Riker, Talantov
ILMN_2714565	*Rrm2*	Mus musculus ribonucleotide reductase M2 polypeptide	1.4	0.002	↑↑	Riker, Talantov
ILMN_1214319	*Gemin6*	Mus musculus gem (nuclear organelle) associated protein 6 (Gemin6), mRNA.	1.4	0.001	↑↑	Riker, Talantov
ILMN_2632712	*Birc5*	Mus musculus baculoviral IAP repeat- containing 5 (Birc5), transcript variant 1, mRNA.	1.4	0.006	↑↑	Riker, Talantov
ILMN_2621422	*Kirrel*	Mus musculus kin of IRRE like (Drosophila) (Kirrel), mRNA.	1.3	0.026	↑↑	Riker, Talantov
ILMN_2484707	*Tyms*	Mus musculus thymidylate synthase (Tyms), mRNA.	1.3	0.013	↑↑	Riker, Talantov
ILMN_1218967	*Kif2c*	Mus musculus kinesin family member 2C (Kif2c), mRNA. XM_986361	1.3	0.015	↑↑	Riker, Talantov
ILMN_1233857	*Mcm6*	Mus musculus minichromosome maintenance deficient 6 (MIS5 homolog, S. pombe) (S. cerevisiae) (Mcm6), mRNA.	1.3	0.012	↑↑	Riker, Talantov
ILMN_2797642	*Ncaph/ Brrn1*	Mus musculus non-SMC condensin I complex, subunit H (Ncaph), mRNA.	1.3	0.013	↑↑	Riker, Talantov
ILMN_2677595	*Ncapg2/Mtb*	Mus musculus non-SMC condensin II complex, subunit G2 (Ncapg2), mRNA.	1.2	0.022	↑↑	Riker, Talantov
ILMN_1245757	*Nans*	Mus musculus N-acetylneuraminic acid synthase (sialic acid synthase) (Nans), mRNA.	1.2	0.027	↑↑	Riker, Talantov
ILMN_2804444	*Pcdh17*	Mus musculus protocadherin 17 (Pcdh17), mRNA.	1.5	0.001	↑↑	Riker
ILMN_2688944	*Htra2/ Prss25*	Mus musculus HtrA serine peptidase 2 (Htra2), nuclear gene encoding mitochondrial protein, mRNA.	1.4	0.001	↑↑	Riker
ILMN_2741050	*Rfc4*	Mus musculus replication factor C (activator 1) 4 (Rfc4), mRNA.	1.4	0.013	↑↑	Riker
ILMN_1257552	*Tars*	Mus musculus threonyl-tRNA synthetase (Tars), mRNA.	1.4	0.008	↑↑	Riker
ILMN_3097131	*Timeless*	Mus musculus timeless homolog (Drosophila) (Timeless), transcript variant 2, mRNA.	1.3	0.013	↑↑	Riker
ILMN_2975640	*Snrpg*	Mus musculus small nuclear ribonucleoprotein polypeptide G (Snrpg), mRNA.	1.2	0.013	↑↑	Riker
ILMN_2711112	*Shcbp1*	Mus musculus Shc SH2-domain binding protein 1 (Shcbp1), mRNA.	1.4	0.001	↑↑	Talantov
ILMN_2607926	*Kdelr2*	Mus musculus KDEL (Lys-Asp-Glu-Leu) endoplasmic reticulum protein retention receptor 2 (Kdelr2), mRNA.	1.4	0.002	↑↑	Talantov
ILMN_2816754	*Ndc80/ Kntc2*	Mus musculus NDC80 homolog, kinetochore complex component (S. cerevisiae) (Ndc80), mRNA.	1.4	0.006	↑↑	Talantov
ILMN_2971845	*Dtymk*	Mus musculus deoxythymidylate kinase (Dtymk), mRNA.	1.4	0.006	↑↑	Talantov
ILMN_2588362	*Cdca8*	Mus musculus cell division cycle associated 8 (Cdca8), mRNA.	1.4	0.003	↑↑	Talantov
ILMN_2934457	*Ran*	Mus musculus RAN, member RAS oncogene family (Ran), mRNA.	1.4	0.004	↑↑	Talantov
ILMN_2999654	*Psmc3ip*	Mus musculus proteasome (prosome, macropain) 26S subunit, ATPase 3, interacting protein (Psmc3ip), mRNA.	1.4	0.003	↑↑	Talantov
ILMN_2598852	*Ranbp1*	Mus musculus RAN binding protein 1 (Ranbp1), mRNA.	1.3	0.007	↑↑	Talantov
ILMN_2639036	*Hspd1*	Mus musculus heat shock protein 1 (chaperonin) (Hspd1), mRNA.	1.3	0.011	↑↑	Talantov
ILMN_2745005	*Gpn3/ MGC14 560*	Mus musculus GPN-loop GTPase 3 (Gpn3), mRNA.	1.3	0.005	↑↑	Talantov
ILMN_2919433	*Cdc45l/Cdc45*	Mus musculus cell division cycle 45 homolog (S. cerevisiae)-like (Cdc45l), mRNA.	1.3	0.043	↑↑	Talantov
ILMN_2749937	*Ncoa6*	Mus musculus nuclear receptor coactivator 6 (Ncoa6), mRNA.	**1.3**	0.011	↑↓	Talantov
ILMN_2605890	*Tk1*	Mus musculus thymidine kinase 1 (Tk1), mRNA.	1.3	0.036	↑↑	Talantov
ILMN_2706882	*Donson*	Mus musculus downstream neighbor of SON (Donson), mRNA.	1.3	0.028	↑↑	Talantov
ILMN_2680648	*Atad2*	Mus musculus ATPase family, AAA domain containing 2 (Atad2), mRNA.	1.3	0.026	↑↑	Talantov
ILMN_2657844	*Cdc2a/ Cdk1*	Mus musculus cell division cycle 2 homolog A (S. pombe) (Cdc2a), mRNA.	1.3	0.021	↑↑	Talantov
ILMN_2608933	*Tcof1*	Mus musculus Treacher Collins Franceschetti syndrome 1, homolog (Tcof1), mRNA.	1.2	0.030	↑↑	Talantov
ILMN_1233065	*Rbmx*	Mus musculus RNA binding motif protein, X chromosome (Rbmx), mRNA.	1.2	0.053	↑↑	Talantov
ILMN_1236574	*Cenpa*	Mus musculus centromere protein A (Cenpa), mRNA.	1.2	0.016	↑↑	Talantov
ILMN_1221067	*Nme1*	Mus musculus non-metastatic cells 1, protein (NM23A) expressed in (Nme1), mRNA.	1.2	0.006	↑↑	Talantov
ILMN_2830661	*Top2a*	Mus musculus topoisomerase (DNA) II alpha (Top2a), mRNA.	1.2	0.025	↑↑	Talantov
ILMN_2936427	*Mcm4*	Mus musculus minichromosome maintenance deficient 4 homolog (S. cerevisiae) (Mcm4), mRNA.	1.2	0.050	↑↑	Talantov
ILMN_2688912	*F10*	Mus musculus coagulation factor X	0.4	0.012	↓↓	Talantov

Forty-one (41) out of 58 genes (from the comparison analysis of our experiment [ten A-I KO vs ten A-I Tg^+/–^ tumors] with human biopsies studies that associated with adverse prognosis) were observed to be differentially regulated (> 2-fold) and statistically significant (*p* ≤ 0.01) in Cutaneous Melanoma vs Normal samples (from either both or one of the two datasets -Riker and Talantov) in Oncomine database. The Riker (Human Genome U133 Plus 2.0 Array, mRNA [40];) has 14 melanoma and 4 normal samples; Talantov (Human Genome U133A Array, mRNA [7];) has 45 melanoma and 7 normal samples.

## DISCUSSION

In this study, we used DNA microarray technology to analyze the tumor microenvironment for transcriptomic patterns that might provide insight as to why the growth and metastasis of B16F10L melanoma was attenuated in apoA-I-expressing mice compared to A-I null animals [16]. We identified 535 transcripts that were differentially expressed in A-I KO and A-I Tg^+/–^ tumors. Of these, 176 transcripts showed statistically significant differences between the two groups (adjusted FDR *p* < 0.05) with at least a 1.2-fold change in expression. We compared our gene expression dataset with published melanoma gene signatures and determined that the expression of 45% of genes associated with poor prognosis in human studies were concordant in tumors from A-I KO relative to A-I Tg^+/–^, thus validating our dataset for mechanistic mining purposes.

Cancer cells adapt their metabolism to fuel their increased proliferation and requisite need for biomass. Figure 3 illustrates that both glucose and glutamine are required for the *de novo* serine/glycine synthesis pathway. Several chemotherapeutic agents target this pathway, underscoring the significance of serine, glycine and one-carbon metabolism for tumor progression [41]. In this study, we identified the *de novo* serine synthesis pathway as a potential target for apoA-I anti-neoplastic activity. *Phgdh*, the gene encoding the first enzyme of this pathway, was the most repressed transcript in tumors recovered from A-I Tg^+/–^ mice relative to A-I KO mice (Table 1B and Supplementary Table 2). This finding suggests that within the tumor cell and tumor microenvironment, apoA-I may inhibit a major metabolic pathway branching off the glycolytic cascade in melanoma.

In humans, the chromosomal locus of *Phgdh* (1p12) is amplified in a subset of melanoma and breast cancers (estrogen receptor-negative), and recent reports using an *in vivo* shRNA library screen and metabolomic profiling support the notion that increased expression of PHGDH promotes tumor growth trending with poor outcome [42, 43]. Importantly, shRNA against *Phgdh* suppressed tumor growth in a human breast xenograft model [43]. Thus, inhibition of PHGDH in cancers overexpressing this key enzyme affects glutamine flux into the TCA cycle as well as serine synthesis leading to growth arrest.

Increased copy number of the *Phgdh* gene in mice led to increased PHGDH expression and promoted the development of melanoma and breast cancer cells [44]. PHGDH was also recently reported to have a non-metabolic role in glioma tumorigenesis via stabilization of the transcription factor FOXM1 [45]. The expression of PHGDH (mRNA and protein), which is absent in normal brain tissue, was increased in gliomas as a direct function of tumor grade, with more aggressive tumors expressing higher levels of this protein [45]. Patient survival (5-year) within grade III and IV groups was 43.3% in the low-PHGDH expression group compared with 18.5% in the high-PHGDH expression group (*p* < 0.001), underscoring PHGDH as a prognostic marker for glioma [45]. Recent reports also identify FOXM1 as being overexpressed in metastatic melanoma [46, 47], and its targeting led to apoptosis in animal models of melanoma and in melanoma cells in culture [48, 49]. In our current study, FOXM1 was not differentially expressed in tumor tissue from A-I KO vs A-I Tg hosts. However, this does not rule out a role for this transcription factor in concert with PHGDH to effect melanoma progression in our animal model.

In this study, we also identified the mevalonate pathway as a potential target for apoA-I anti-tumor activity (Figure 5). Transcripts for five enzymes in this pathway were downregulated in tumors from A-I Tg^+/–^ mice (Figure 5, enzymes highlighted in box). The mevalonate pathway feeds into several growth promoting pathways including cell signaling, lipid metabolism, cell structure, and nutrient levels [50], and has also been identified as a positive regulator of YAP and TAZ proto-oncogenes, which are targets of the Hippo tumor-suppressor pathway [51, 52]. The transcriptional expression of YAP is driven by the ETS transcript factor GA-binding protein (GABP) [53]. Interestingly, GABP DNA-binding elements were significantly enriched in the promoter region of genes down regulated in this study’s dataset (data not shown). Inactivating mutations in the tumor suppressor gene p53 has also been correlated with increased expression of mevalonate pathway genes [54]. In cancers expressing mutant p53, the latter acts as a coactivator of SREBP (sterol regulatory element-binding protein), and serves as the positive master transcription factor for enzymes of the mevalonate pathway and for fatty acid synthesis [55].

The YAP/TAZ proto-oncogenes promote cancer stemness and metastatic potential [56, 57]. Inhibition of the mevalonate pathway by statins leads to transcriptional repression of the YAP-dependent pro-metastatic gene RHAMM in breast cancer cells expressing mutant p53 [58, 59]. Four (*Sqle, Cyp51, Hsd17b7, and Dhcr24*) of the five negatively regulated genes in this study’s dataset are in the sterol synthesis arm of mevalonate pathway (Figure 5). The fifth, *Mvd,* which encodes mevalonate-5-pyrophosphate decarboxylase, is upstream of the branching point (farnesyl-diphosphate; farnesyl-PP in Figure 5) in the pathway. Thus, inhibition of MVD is likely to have a larger impact, as it affects all branches of the mevalonate pathway. The anti-tumor effect of statin therapy via inhibition of the mevalonate pathway appears to be restricted to cancers expressing mutant p53, and in breast cancers is further restricted to the hormone receptor negative subtypes (ER-/PR-) [55]. B16F10, the murine melanoma cell line used in this study expresses mutant p53 [60]. Recently, the ATP-binding cassette transporter A1 (ABCA1), which facilitates the active efflux of lipids and cholesterol to apoA-I thereby initiating HDL biogenesis, was identified as a transcriptional target of wild-type p53, and mediator of p53 inhibition of the mevalonate pathway via SREBP-2 maturation, in a murine model of liver cancer [61]. Whether apoA-I mediates mevalonate pathway inhibition through the p53/SREBP arm in melanoma remains to be determined.

Supplementary Table 2 lists genes that were downregulated in tumors from A-I Tg^+/–^ animals (relative to A-I KO) and encode proteins that included Dicer, Adamts1 and Cdc20. Dicer, an RNase III family member, processes precursor miRNAs to smaller active species. In melanoma, miRNA have been shown to be deregulated in cell lines [62–64], metastatic lesions ([65], and primary clinical samples [66]. Increased Dicer expression was correlated with progression of melanoma from common melanocytic nevus to invasive melanoma [67]. The extracellular protease Adamts1 (a disintegrin and metalloproteinase with thrombospondin motifs 1) has been implicated in promoting cancer [68], and stroma-derived Adamts1 was recently shown to drive both the growth and metastasis of melanoma [69]. ApoA-I likely inhibits expression of Adamts1 in both tumor and stromal cells, though the mechanism remains to be elucidated.

Cdc20, a key cell cycle regulator of spindle checkpoint and mitotic exit, is suppressed by p53 tumor suppressor protein and is frequently up regulated in cancers due to inactivation of p53 [70]. Recently, systemic delivery of *Cdc20* siRNA by intraperitoneal injection was shown to inhibit B16F10 melanoma growth in tumor bearing mice [71, 72] and Cdc20 has been proposed as a cancer therapeutic target [72]. In the present study, two separate probes specific for *Cdc20* showed statistically significant reduced expression in 11 separate tumors resected from A-I Tg^+/–^ animals relative to A-I KO (Supplementary Table 2).

The emerging theme in this study is that in animals expressing apoA-I, two major metabolic pathways, namely the mevalonate and serine synthesis pathways, are inhibited within the melanoma tumor microenvironment. The products of these pathways provide molecules indispensable for synthesis of essential lipids, proteins, and nucleic acids, and inhibitors of the mevalonate pathway have been shown to suppress tumor development and metastasis in both mouse [73, 74] and human melanoma cells [75, 76]. The mechanism (s) by which increased levels of apoA-I in host animals engenders this inhibition remains to be elucidated. The apoA-I receptor ABCA1 was shown to mediate inhibition of the mevalonate pathway via SREBP-2 maturation [61]. ABCA1, ABCG1 (HDL receptor) and SRBP-1 (HDL receptor) are candidates for further investigation as potential mediators of apoA-I anti-tumor activity in B16F10 melanoma.

Finally, recent studies with statin treatment of melanoma cells concluded that inhibition of the mevalonate pathway stimulates melanoma immunogenicity [76] and leads to increased adaptive [77, 78] and innate [75] immune response against the tumor [76]. Our current study comparing A-I Tg^+/–^ and A-I KO tumor bearing mice suggest that apoA-I may target cancer metabolism. Importantly, we observed an inverse correlation between circulating HDL-c and transcript levels of *Phgdh* and five enzymes of the mevalonate pathway (*Mvd*, *Sqle, Cyp51, Hsd17b7,* and *Dhcr24*) in tumor bed, suggestive of an inhibitory role for apoA-I/HDL in cancer cell metabolism (Figure 6). Thus, apoA-I has emerged as a viable therapeutic participant at the crossroads between cardiovascular disease and cancer, considering the growing evidence of biological and mechanistic overlap between these two main causes of mortality worldwide [79–81].

## MATERIALS AND METHODS

All chemicals were from Sigma Chemical (St. Louis, MO) and all solvents were HPLC grade unless otherwise indicated.

### Mice

All mouse studies were performed under approved Institutional Animal Care and Use Committee protocols at the Cleveland Clinic. C57BL/6J (wild type (WT) mice), A-I KO, A-I Tg^+/+^, mice were purchased from Jackson Laboratories and bred at Cleveland Clinic’s Biological Research Unit (BRU).

### Plasma HDL-c levels

Blood was drawn at the time of tumor resection by cardiac puncture from anaesthetized (300 mg/kg ketamine (Ketaset (Ketamine HCl Injection, USP) Zoetis 100 mg/mL) plus 30 mg/kg xylazine (AnaSed Injection Akorn Animal Health 100 mg/mL NDC 59399-111-50) mice on day-7 post tumor inoculation. Plasma (EDTA) was separated and diluted in saline for HDL-c measurements on an Architect ci8200 (Abbott Diagnostics, Abbot Park, IL) using the Ultra HDL assay kit (3K33-20).

### Tumor cell line

Mouse tumor cell lines B16F10 melanoma were obtained from American Type Culture Collection (ATTC, Bethesda, MD) and cultured in DMEM supplemented with 10% heat-inactivated fetal calf serum (FCS), 2 mM L-glutamine and antibiotic/antimycotic (Invitrogen, Grand Island, NY) at 37°C and 5% CO_2_ in a humidified atmosphere. B16F10L was isolated from lung metastasis in WT C57BL/6J and a variant expressing luciferase (firefly, *Photinus pyralis*) was engineered by cotransfecting B16F10L cells with pKCPIRlucBGH5.3 (kind gift from Yan Xu, Indiana University School of Medicine) and pcDNA3 (Invitrogen, 3:1 ratio) using Lipofectamine (Invitrogen) and selected with 1.1 mg/mL G418 (Invitrogen) over 3 wk. Pools of luciferase expressing tumor cells were used for the study.

### RNA isolation from tumor tissue

Eight to ten (8–10) week male and female animals were inoculated subcutaneously on both flanks with 10^5^ B16F10L tumor cells per site (2 sites/flank). Tumor tissue from one flank was resected on day 7 post inoculation and stored in RNA later. Tumor tissue (30 mg) was homogenized with zirconium oxide beads using the Bullet blender tissue homogenizer (Next Advance, Troy, NY, USA) and total RNA was prepared (RNeasy Mini kit, Qiagen, Valencia, CA) with on column DNase treatment (Cat# 79254, Qiagen).

### Gene expression analysis

Global gene expression patterns were obtained using Illumina gene chips. Total RNA, prepared as described above, was amplified and biotinylated for hybridization to Illumina Murine Beadchips (mouseRef-8 v.2, Illumina, Inc., San Diego, CA) using Illumina TotalPrep RNA Amplification Kit (AMIL1791, Applied Biosystems, Life Technologies) according to the manufacturer’s protocol. Arrays were scanned in an Illumina Bead Station and the images were processed using Illumina Bead Studio software. The complete microarray data have been deposited in the Gene Express Omnibus (GEO) database (GEO: GSE137532). Gene expression profiles were compared using unsupervised hierarchical clustering analysis.

### Validation of mouse gene expression set with studies with human melanoma

Gene expression data were analyzed in GenomeStudio. Raw data were quantile-normalized, background corrected, and filtered with a detection *p* < 0.05 and a 1.2-fold-change in expression between A-I KO and A-I Tg^+/–^. One-hundred-thirty (130) human genes previously identified as part of a gene signature associated with poor prognosis in melanoma [8] were compared with genes identified in this study from the differential analysis of day 7 B16F10L melanoma tumors with respect to fold-change (A-I KO/A-I Tg^+/–^) in expression signal normalized to beta-2-microglobulin with a *p*-value cut off < 0.05 (Student’s *t*-test). The human microarray study identified the expression profile of genes associated with 4-year distant metastasis-free survival, metastasis, or death among 58 patients with 4-yr follow up [8]. Of the 250 human genes identified, only 130 were eligible for comparison in our current study.

### Statistical analysis of gene expression data: unbiased mining for significant genes with differential regulation in A-I Tg^+/-^vs A-I KO mouse tumors

Raw data were read in GenomeStudio and exported for further data processing and analysis in R software (http://www.r-project.org/). Analyses were done on the data after force-positive background correction, log2 transformation, and quantile normalization. Quality control analysis was performed. Comparisons were made to check differences between each of two groups Tg^+/–^ vs KO and WT vs KO. The comparisons were performed using linear models. Empirical Bayes and other shrinkage methods were used to borrow information across genes. FDR (Benjamini & Hochberg correction,) adjusted *p*-values were calculated to account for multi-testing. R limma package was used. Genes are significantly expressed if FDR adjusted *p*< 0.05. Volcano plots were made to show both the statistical significance (y axis, -log10(*p*- value), the higher the number is, the more significant the test is) and biological significance (x axis, log2(Fold-Change), the further right from the zero point, the more upregulated, the further left, the more down regulated). Heatmap and dendrogram from unsupervised hierarchical clustering analysis were plotted.

### Bioinformatics analyses

Functional annotation of statistically significant list of genes, in terms of how they relate to key processes or pathways of interest, were obtained using publicly available genome annotation tools and pathway databases such as KEGG, DAVID, GOTermMapper, GOTermFinder, Mouse Genome Informatics, and the commercial pathway analysis tools Metacore (Metacore™, https://portal.genego.com/) and Ingenuity Pathway Analysis (Ingenuity^®^ Systems, http://www.ingenuity.com/). Commercial pathway databases were used in addition to publicly available databases because they provided more comprehensive functional information for these genes, not only in the context of physiological processes and pathway but also their molecular interaction networks, drug targets, diseases, and other vital information. For genes that did not map to any known pathways, a map of proteins interacting upstream or downstream of these genes were obtained from these commercially available pathway analysis. The data discussed in this publication have been deposited in NCBI′s Gene Expression Omnibus (Edgar *et al*., 2002) and are accessible through GEO Series accession number GSE137532, https://www.ncbi.nlm.nih.gov/geo/query/acc.cgi?acc= GSE137532).

## SUPPLEMENTARY MATERIALS



## Abbreviations

3PG: 3-phosphoglycerate
A-I KO: apoA-I knockout
A-I Tg^+/–^: apoA-I transgenic (heterozygous)
ABCA1: ATP binding cassette subfamily A member 1
ABCG1: ATP-binding cassette (ABC) G-1
Adamts1: a disintegrin and metalloproteinase with thrombospondin type 1 motif
aKG: alpha-ketoglutarate
ApoA-I: apolipoprotein A-I
Cdc20: cell division cycle 20 homolog
Cyp51: sterol 14α-demethylase cytochrome P450
Dhcr24: 24-dehydrocholesterol reductase
F10: vitamin K-dependent coagulation factor X
FDR: false discovery rate
FOXM1: forkhead box protein M1
GABP: GA-binding protein
Glu: glutamate
HDL: high density lipoprotein
HDL-c: high density lipoprotein-cholesterol
HMG-CoA: 3-hydroxy-3-methyl-glutaryl-co-enzyme A
Hsd17b7: 3-keto-steroid reductase
IPA: Ingenuity pathway Analysis
MVD: mevalonate-5-pyrophosphate decarboxylase
Ncoa6: nuclear receptor coactivator 6
P-PYR: 3-phospho-hydroxypyruvate
P-serine: phosphoserine
Phgdh: phosphoglycerate dehydrogenase
PSAT1: phosphoserine aminotransferase
PSPH: phosphoserine phosphatase
RHAMM: hyaluronan-mediated motility receptor
Sqle: squalene epoxidase
SREBP: sterol regulatory element-binding protein
TAZ: tafazzin
TCA cycle: the citric acid cycle
WT: wild type
YAP: yes-associated protein
