# Supplementary material for: Apolipoprotein A-I anti-tumor activity targets cancer cell metabolism
Source: Oncotarget. 2020 May 12;11(19):1777–96. doi: 10.18632/oncotarget.27590 (PMC7233810; doi:10.18632/oncotarget.27590)
Supplement: Supplementary file 1 [file oncotarget-11-1777-s001.pdf]

Apolipoprotein A-I anti-tumor activity targets cancer cell metabolism

SUPPLEMENTARY MATERIALS

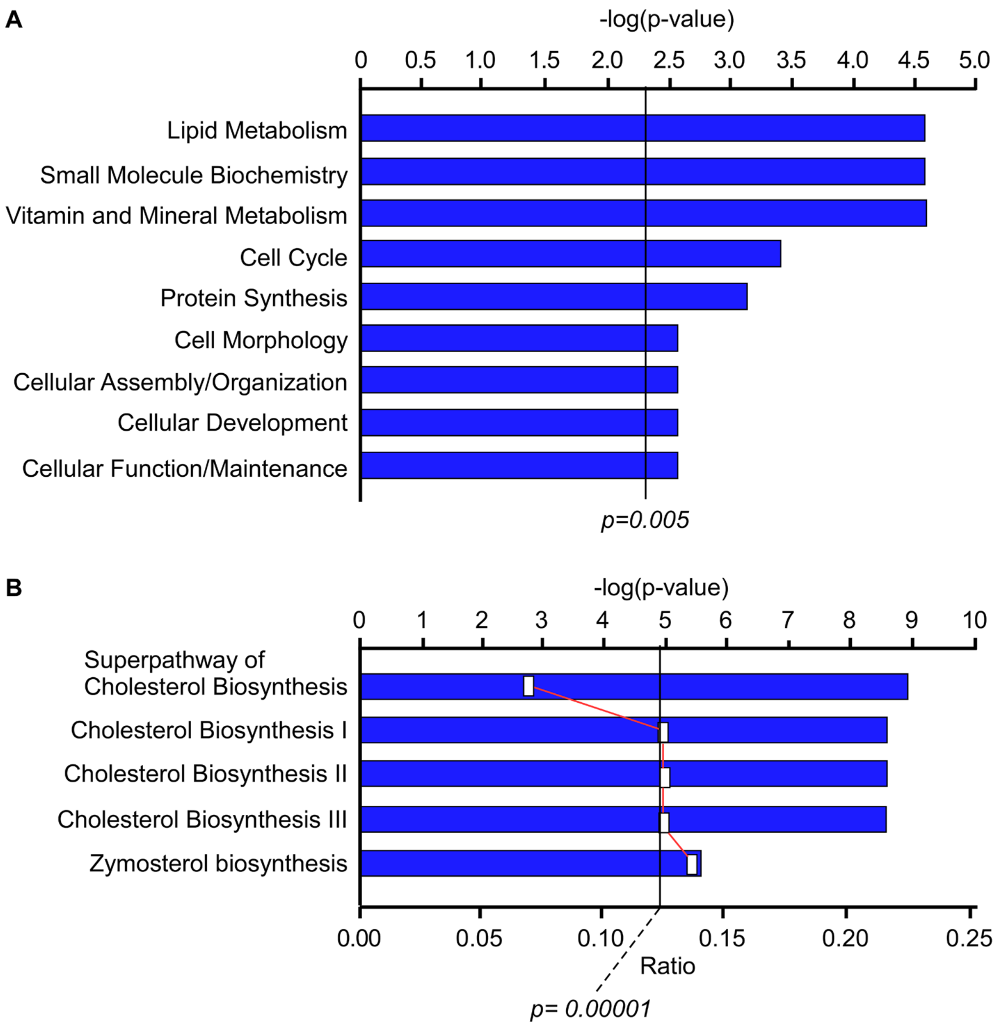

Supplementary Figure 1: (**A**) Lipid metabolism is the top molecular and cellular function, (**B**) Cholesterol biosynthesis is the top canonical pathway mapped to genes down-regulated in primary melanoma tumors from A-I Tg$^{+/-}$ mice relative to A-I KO. Genes listed in Supplementary Table 2 were analyzed in IPA (Ingenuity Pathway Analysis) to generate biological and functional relationships based on published literature. Scoring Method: Fisher's Exact Test, *p*-value 0.005. See Supplementary Table 3 for list of genes in molecular & cellular functions (A) and canonical pathways (B).

Supplementary Table 1: List of significant probes (FDR adjusted p < 0.05) up-regulated (fold change cut off > 1.2) in tumors from A-I Tg$^{+/-}$ relative to A-I KO mice

Probe_ID	Accession no.	Gene Symbol	Name	FC	adj.*p*- Value
ILMN_1251748	NM_139200.4	*Cytip*	cytohesin 1 interacting protein	1.83	0.040
ILMN_1253182	NM_010474.1	*Hs3st1*	heparan sulfate (glucosamine) 3-O-sulfotransferase 1	1.81	0.012
ILMN_2622983	NM_013642.2	*Dusp1*	dual specificity phosphatase 1	1.67	0.033
ILMN_3161601	NM_009221.2	*Snca*	synuclein, alpha (non A4 component of amyloid precursor)	1.54	0.011
ILMN_2705166	NM_145933.3	*St6gal1*	ST6 beta-galactosamide alpha-2,6-sialyltranferase 1	1.49	0.012
ILMN_2722732	NM_011157.2	*Srgn*	serglycin	1.46	0.015
ILMN_3136638	NM_009221.2	*Snca*	synuclein, alpha (non A4 component of amyloid precursor)	1.44	0.011
ILMN_1254031	NM_010638.4	*Klf9*	Kruppel-like factor 9	1.43	0.008
ILMN_3161105	NM_001033476.1	*Ahnak2*	AHNAK nucleoprotein 2	1.42	0.047
ILMN_2829594	NM_010479.2	*Hspa1a*	heat shock 70kDa protein 1A	1.40	0.006
ILMN_2754985	NM_009344.1	*Phlda1*	pleckstrin homology-like domain, family A, member 1	1.40	0.006
ILMN_2824971	NM_001004761.1	*Gpr158*	G protein-coupled receptor 158	1.39	0.011
ILMN_2896314	NM_015732.3	*Axin2*	axin 2	1.38	0.004
ILMN_1233064	NM_183417.2	*Cdk2*	cyclin-dependent kinase 2	1.37	0.009
ILMN_2892441	NM_010357.1	*Gsta4*	glutathione S-transferase alpha 4	1.37	0.029
ILMN_2831799	NM_019517.2	*Bace2*	beta-site APP-cleaving enzyme 2	1.36	0.013
ILMN_2623280	NM_011019.1	*Osmr*	oncostatin M receptor	1.36	0.008
ILMN_2522236	NM_011661.3	*Tyr*	tyrosinase	1.36	0.039
ILMN_3114585	NM_001039150.1	*Cd44*	CD44 molecule	1.35	0.002
ILMN_1252202	NM_009397.2	*Tnfaip3*	tumor necrosis factor, alpha-induced protein 3	1.34	0.030
ILMN_2612895	NM_013484.1	*C2*	complement component 2	1.34	0.014
ILMN_2680415	NM_172537.2	*Sema6d*	Semaphorin	1.34	0.028
ILMN_1246153	NM_133362.2	*Erdr1*	erythroid differentiation regulator 1	1.34	0.017
ILMN_1240323	NM_018808.1	*Dnajb1*	DnaJ (Hsp40) homolog, subfamily B, member 1	1.34	0.001
ILMN_2813484	NM_011065.2	*Per1*	period circadian clock 1	1.33	0.015
ILMN_2774690	XM_001004685.1	*LOC677317*	similar to NADP-dependent malic enzyme (NADP-ME) (Malic enzyme 1)	1.33	0.049
ILMN_2734181	NM_019811.3	*Acss2*	acyl-CoA synthetase short-chain family member 2	1.32	0.026
ILMN_1226157	NM_181585.5	*Pik3r3*	phosphoinositide-3-kinase, regulatory subunit 3 (gamma)	1.32	0.042
ILMN_2746556	NM_015814.2	*Dkk3*	dickkopf 3 homolog (Xenopus laevis)	1.31	0.022
ILMN_3073563	NM_001001884.1	*C230021P08Rik*	Nckap5l NCK-associated protein 5-like	1.31	0.015
ILMN_2776619	NM_008520.2	*Ltbp3*	latent transforming growth factor beta binding protein 3	1.30	0.012
ILMN_1258600	XM_001481024.1	*LOC100043671*	LOC100043671 hypothetical protein	1.30	0.040
ILMN_2870672	NM_010180.1	*Fbln1*	fibulin 1	1.30	0.015
ILMN_1241965	NM_008601.2	*Mitf*	microphthalmia-associated transcription factor	1.29	0.037
ILMN_2776909	NM_008393	*Irx3*	iroquois homeobox 3	1.28	0.022
ILMN_1227951	NM_133219.1	*Gcnt2*	glucosaminyl (N-acetyl) transferase 2, I-branching enzyme	1.28	0.037
ILMN_2976829	NM_013506.1	*Eif4a2*	eukaryotic translation initiation factor 4A2	1.28	0.031
ILMN_2431871	XM_125493	*Fbxo30*	F-box protein 30	1.27	0.014
ILMN_2942011	NM_028783.2	*Robo4*	roundabout, axon guidance receptor, homolog 4 (Drosophila)	1.27	0.040
ILMN_1242622	NM_013498.1	*Crem*	cAMP responsive element modulator	1.27	0.010
ILMN_2630459	NM_009911.2	*Cxcr4*	chemokine (C-X-C motif) receptor 4	1.27	0.011
ILMN_2954474	NM_015744.1	*Enpp2*	ectonucleotide pyrophosphatase/phosphodiesterase 2	1.27	0.043
ILMN_1252210	NM_028314.1	*2700097O09Rik*	RIKEN cDNA 2700097O09 gene	1.27	0.026
ILMN_2720083	NM_019517.3	*Bace2*	beta-site APP-cleaving enzyme 2	1.26	0.026
ILMN_1241142	NM_001040682.1	*Clmn*	calmin	1.26	0.050
ILMN_2877958	NM_009855.2	*Cd80*	Cd80 costimulatory molecule	1.25	0.025
ILMN_1228501	NM_020507.3	*Tob2*	member of BTG/Tob anti-proliferative family	1.25	0.009
ILMN_2733887	NM_021462.3	*Mknk2*	MAP kinase-interacting kinase 2	1.24	0.026
ILMN_1245813	NM_175352.4	*Unc119b*	SH3 ligand Uncoordinated 119	1.24	0.007
ILMN_3005873	NM_019972.2	*Sort1*	sortilin	1.24	0.005

ILMN_3138743	NM_028817.2	*Acsl3*	acyl-coenzymeA (CoA) synthetase isoforms 3	1.23	0.026
ILMN_1245815	NM_173755.3	*Ube2o*	E2 ubiquitin-conjugating (UBC) enzyme	1.23	0.019
ILMN_1218934	NM_025654.2	*Rdm1*	RAD52 Motif 1	1.23	0.019
ILMN_2928498	NM_029946.3	*Efcab6*	EF- hand calcium binding domain 6	1.22	0.004
ILMN_1222132	NM_198292.3	*Tex2*	Tex2 testis expressed gene 2	1.22	0.018
ILMN_2792089	NM_011480.1	*Srebf1*	Sterol regulatory element-binding protein 1	1.22	0.004
ILMN_2653725	XM_001476835.1	*LOC100046802*	similar to Inhbb protein	1.22	0.022
ILMN_2690256	NM_133748.1	*Insig2*	insulin induced gene 2	1.22	0.009
ILMN_2668849	NM_027230.3	*Prkcbp1*	zinc finger, MYND-type containing 8	1.21	0.010
ILMN_3160688	NM_001033159.2	*Zfp597*	zinc finger protein 597	1.21	0.009
ILMN_2650603	NM_018826.2	*Irx5*	iroquois homeobox 5	1.21	0.032
ILMN_2798797	NM_026313.1	*3300001P08Rik*	LUC7-like 3	1.21	0.009
ILMN_2740020	NM_010838.2	*Mapt*	microtubule-associated protein tau	1.21	0.026
ILMN_2737758	NM_011218.1	*Ptprs*	protein tyrosine phosphatase, receptor type, S	1.21	0.031
ILMN_2725370	NM_009582.3	*Map3k12*	mitogen-activated protein kinase kinase kinase 12	1.21	0.012
ILMN_2627441	NM_023324.2	*Peli1*	pellino E3 ubiquitin protein ligase 1	1.21	0.033
ILMN_2612448	NM_133957.2	*Nfat5*	nuclear factor of activated T-cells 5, tonicity-responsive	1.21	0.033
ILMN_1243812	NM_031256.2	*Plekha3*	pleckstrin homology domain containing, family A	1.21	0.050
ILMN_2867789	NM_001037923.1	*EG624866*	predicted gene, EG624866	1.20	0.010
ILMN_2728985	NM_010180.1	*Fbln1*	fibulin 1	1.20	0.036
ILMN_2652757	NM_134255.2	*Elovl5*	ELOVL fatty acid elongase 5	1.20	0.022

Eleven separate primary tumors from eleven A-I $Tg^{+/-}$ or A-I KO mice were subjected to differential gene expression analysis. B16F10L melanoma homograft grown in C57BL/6 mice deficient in apoA-I (A-I KO) or expressing human apoA-I (A-I $Tg^{+/-}$) were resected 7 days after inoculation and processed for RNA, microarray and data analysis as described in Methods.

Supplementary Table 2: List of significant probes (FDR adjusted *p* < 0.05) down-regulated (fold change cut off > 1.2) in tumors from A-I Tg$^{+/-}$ relative to A-I KO mice

Probe_ID	Accession no.	Symbol	Name	FC	adj. *p*- Value
ILMN_2790181	NM_016966.3	*Phgdh*	phosphoglycerate dehydrogenase	1.57	0.027
ILMN_3081854	NM_001025245.1	*Mbp*	myelin basic protein	1.48	0.023
ILMN_2600348	NM_009270.3	*Sqle*	squalene epoxidase	1.47	0.004
ILMN_2634905	NM_007994.3	*Fbp2*	fructose-1,6-bisphosphatase 2	1.45	0.022
ILMN_2772274	NM_001079694.1	*Sfrs5*	serine/arginine-rich splicing factor 5	1.37	0.002
ILMN_2688075	NM_020010.2	*Cyp51*	cytochrome P450, family 51	1.35	0.005
ILMN_3148662	NM_001079695.1	*Sfrs5*	serine/arginine-rich splicing factor 5	1.35	0.009
ILMN_2712557	NM_024439.3	*H47*	histocompatibility 47 Gene	1.34	0.032
ILMN_2996877	NM_010063.1	*Dync1i1*	dynein cytoplasmic 1 intermediate chain 1	1.34	0.013
ILMN_2700292	NM_010376.3	*H13*	histocompatibility 13	1.33	0.011
ILMN_2630641	NM_009272.4	*Srm*	spermidine synthase	1.33	0.009
ILMN_2737163	NM_009270.3	*Sqle*	squalene epoxidase	1.32	0.004
ILMN_2761594	NM_007705.2	*Cirbp*	cold inducible RNA binding protein	1.32	0.009
ILMN_2660414	NM_025442.3	*Alg5*	ALG5, dolichyl-phosphate beta-glucosyltransferase	1.32	0.018
ILMN_3086899	NM_198104.2	*Tcte3*	t-complex-associated-testis-expressed 3	1.32	0.004
ILMN_2823778	NM_025436.1	*Sc4mol*	(testis meiosis-activating sterol/sterol C4-methyl oxidase–like)	1.32	0.028
ILMN_2642417	NM_008590.1	*Mest*	mesoderm specific transcript	1.31	0.048
ILMN_1247916	NM_144862.3	*Lims2*	LIM and senescent cell antigen-like domains 2	1.31	0.044
ILMN_2739825	NM_010063.1	*Dync1i1*	dynein cytoplasmic 1 intermediate chain 1	1.31	0.032
ILMN_1229529	NM_010476.3	*Hsd17b7*	hydroxysteroid (17-beta) dehydrogenase 7	1.30	0.004
ILMN_2606693	NM_153526.4	*Insig1*	insulin induced gene 1	1.30	0.031
ILMN_1238654	NM_027352.3	*Gorasp2*	golgi reassembly stacking protein 2	1.30	0.041
ILMN_2737200	NM_010777.3	*Mbp*	myelin basic protein	1.29	0.023
ILMN_1234453	XM_001480197.1	*LOC100043257*	similar to RNA binding motif protein 3	1.29	0.008
ILMN_2720674	NM_023377.4	*Stard5*	StAR-related lipid transfer protein 5	1.29	0.032
ILMN_2612350	NM_026959.2	*Stx18*	syntaxin 18	1.29	0.026
ILMN_1245272	NM_153526.4	*Insig1*	insulin induced gene 1	1.27	0.047
ILMN_2654952	XM_001475189.1	*LOC100040592*	similar to Hmgcs1 protein, transcript variant 1	1.27	0.004
ILMN_2787738	NM_144918.1	*Smyd5*	SET and MYND domain containing 5	1.27	0.040
ILMN_1237990	NM_178389.3	*Gale*	galactose-4-epimerase, UDP	1.27	0.029
ILMN_2776231	NM_019929.3	*Sumo3*	SMT3 suppressor of mif two 3 homolog 3	1.27	0.010
ILMN_2948296	NM_138684.2	*Wfdc12*	WAP four-disulfide core domain 12	1.26	0.047
ILMN_2949275	NM_177684.2	*Zfp637*	zinc finger protein 637	1.26	0.004
ILMN_2747480	NM_026130.1	*Srpr*	signal recognition particle receptor	1.26	0.044
ILMN_2678127	NM_080563.3	*Rnf144a*	ring finger protein 144A	1.26	0.032
ILMN_2698767	NM_023223.1	*Cdc20*	cell division cycle 20 homolog (S. cerevisiae)	1.26	0.033
ILMN_2804444	NM_001013753.1	*Pcdh17*	protocadherin 17	1.25	0.048
ILMN_2907721	NM_153596.1	*Tmem17*	transmembrane protein 17	1.25	0.009
ILMN_2614853	NM_177374.2	*6720458F09Rik*	RIKEN cDNA 6720458F09 gene	1.25	0.020
ILMN_2678828	NM_181582.3	*Eif5a*	eukaryotic translation initiation factor 5A	1.25	0.031
ILMN_3077377	NM_011710.2	*Wars*	tryptophanyl-tRNA synthetase	1.25	0.033
ILMN_2738837	NM_022988.2	*Nif3l1*	Ngg1 interacting factor 3-like 1 (S. pombe)	1.25	0.036
ILMN_1246694	NM_026824.4	*Dus1l*	dihydrouridine synthase 1-like (S. cerevisiae)	1.25	0.010
ILMN_1240445	NM_133774.4	*Stard4*	StAR-related lipid transfer (START) domain containing 4	1.25	0.033
ILMN_2776230	NM_019929.1	*Sumo3*	SMT3 suppressor of mif two 3 homolog 3	1.24	0.025
ILMN_2918114	NM_009687.1	*Apex1*	apurinic/apyrimidinic endonuclease 1	1.24	0.037
ILMN_1226334	NM_025878	*Mrps18b*	mitochondrial ribosomal protein S18B	1.24	0.046
ILMN_1221243	NM_028493.2	*Rhobtb3*	Rho-related BTB domain containing 3	1.24	0.027
ILMN_2645275	NM_138656.1	*Mvd*	mevalonate (diphospho) decarboxylase	1.24	0.015
ILMN_2922728	NM_028456.1	*Rwdd3*	RWD domain containing 3	1.24	0.013
ILMN_2781966	NM_001008705.1	*Bud31*	BUD31 homolog (yeast)	1.23	0.029

ILMN_3022428	NM_001080129.1	*Tmpo*	thymopoietin	1.23	0.018
ILMN_1223547	NM_026553.3	*Yif1a*	Yip1 interacting factor homolog A (S. cerevisiae)	1.23	0.022
ILMN_1255110	NM_016776.2	*Mybbp1a*	MYB binding protein (P160) 1a	1.23	0.020
ILMN_2809611	NM_009272.2	*Srm*	spermidine synthase	1.23	0.023
ILMN_2717387	NM_025386.2	*Fbxo36*	F-box protein 36	1.23	0.022
ILMN_1222503	NM_025442.3	*Alg5*	ALG5, dolichyl-phosphate beta-glucosyltransferase	1.23	0.044
ILMN_2944939	NM_133926.2	*Camk1*	calcium/calmodulin-dependent protein kinase I	1.23	0.019
ILMN_2691493	NM_020491.4	*Sssca1*	Sjogren’s syndrome/scleroderma autoantigen 1	1.23	0.009
ILMN_2629993	NM_144525.3	*Tmem214*	transmembrane protein 214	1.23	0.048
ILMN_2984434	NM_145431.1	*Nle1*	notchless homolog 1	1.23	0.008
ILMN_2693535	NM_134010.2	*Nup107*	nucleoporin 107	1.23	0.007
ILMN_2683621	NM_016703.1	*Preb*	prolactin regulatory element binding	1.23	0.029
ILMN_2629648	NM_025880.3	*2410002F23Rik*	RIKEN cDNA 2410002F23	1.22	0.023
ILMN_2761082	NM_009621.3	*Adamts1*	a disintegrin-like and metallopeptidase (reprolysin type) with thrombospondin type 1 motif, 1	1.22	0.037
ILMN_2484918	NM_016862.3	*Vti1a*	vesicle transport through interaction with t-SNAREs 1A	1.22	0.005
ILMN_2602581	NM_025508.3	*Gmpr*	guanosine monophosphate reductase	1.22	0.037
ILMN_2851040	NM_008563.2	*Mcm3*	minichromosome maintenance deficient 3 (S. cerevisiae)	1.22	0.031
ILMN_2650775	NM_133800.3	*Nol12*	nucleolar protein 12	1.22	0.030
ILMN_2629748	NM_007842.1	*Dhx9*	DEAH (Asp-Glu-Ala-His) box polypeptide 9	1.22	0.023
ILMN_1215331	NM_148948.2	*Dicer1*	dicer 1, ribonuclease type III	1.22	0.010
ILMN_1247582	NM_015782.2	*Snrpa*	small nuclear ribonucleoprotein polypeptide A	1.22	0.046
ILMN_1245860	NM_024193.2	*Nol5a*	Nucleolar protein 5A	1.22	0.026
ILMN_1220842	NM_001033794.1	*C85627*	expressed sequence C85627	1.22	0.024
ILMN_2695528	NM_134020.1	*Tmed4*	transmembrane emp24 protein transport domain containing 4	1.22	0.024
ILMN_2482555	NM_152234.1	*Ubqln1*	ubiquilin 1 (Ubqln1), transcript variant 2	1.22	0.022
ILMN_2607369	NM_021313.1	*Rnf25*	ring finger protein 25	1.22	0.007
ILMN_2685811	NM_011900.3	*Mpdu1*	mannose-P-dolichol utilization defect 1	1.22	0.045
ILMN_1217262	NM_024231.2	*Zfpl1*	zinc finger like protein 1	1.21	0.047
ILMN_2667551	NM_175399.4	*Exosc4*	exosome component 4	1.21	0.004
ILMN_3009910	NM_026453.1	*Rbm13*	RNA binding motif protein 13	1.21	0.008
ILMN_1214974	NM_010744.1	*Il1rl1l/Tmed1*	Interleukin-1 receptor-like 1 ligand/transmembrane emp24 domain containing 1	1.21	0.037
ILMN_2753226	NM_009013	*Rad51ap1*	RAD51 associated protein 1	1.21	0.032
ILMN_2867899	NM_023223.1	*Cdc20*	cell division cycle 20 homolog (S. cerevisiae)	1.21	0.026
ILMN_3003242	NM_008622.1	*Mpv17*	Mpv17 transgene, kidney disease mutant	1.21	0.041
ILMN_2619639	NM_153577.2	*AI428936*	Nesprin-4	1.21	0.010
ILMN_2695526	NM_134020.1	*Tmed4*	transmembrane emp24 protein transport domain containing 4	1.21	0.029
ILMN_1244161	NM_019731.1	*Nme4*	NME/NM23 nucleoside diphosphate kinase 4	1.21	0.005
ILMN_2620106	NM_053272	*Dhcr24*	24-dehydrocholesterol reductase	1.21	0.019
ILMN_3143506	NM_019422.2	*Elovl1*	elongation of very long chain fatty acids transcript variant 2	1.21	0.030
ILMN_1215755	NM_153419.1	*Grwd1*	glutamate-rich WD repeat containing 1	1.21	0.004
ILMN_2890357	NM_172145.2	*2610027C15Rik*	RIKEN cDNA 2610027C15 gene	1.21	0.042
ILMN_2642052	XR_005252.1	*3010003L21Rik*	RIKEN cDNA 3010003L21 gene	1.21	0.024
ILMN_1223819	NM_021537.2	*Stk25*	serine/threonine kinase 25 (yeast)	1.20	0.041
ILMN_1241864	NM_027239.1	*1810065E05Rik*	RIKEN cDNA 1810065E05 gene	1.20	0.037
ILMN_2721208	NM_144898.1	*Msto1*	misato homolog 1 (Drosophila)	1.20	0.044
ILMN_1236100	NM_028456.1	*Rwdd3*	RWD domain containing 3	1.20	0.018
ILMN_2939503	NM_153392.1	*Ttc39a*	tetratricopeptide repeat domain 39A	1.20	0.028
ILMN_2662509	NM_010175.4	*Fadd*	Fas (TNFRSF6)-associated via death domain	1.20	0.004
ILMN_1225544	NM_031196.2	*Slc19a1*	solute carrier family 19 (folate transporter), member 1	1.20	0.014
ILMN_1235751	NM_026824.4	*Dus1l*	dihydrouridine synthase 1-like (S. cerevisiae)	1.20	0.026
ILMN_2683414	NM_033568.2	*Snf8*	SNF8, ESCRT-II complex subunit, homolog (S. cerevisiae)	1.20	0.041
ILMN_2987339	NM_016776.2	*Mybbp1a*	MYB binding protein (P160) 1a	1.20	0.018
ILMN_1223126	NM_010175.4	*Fadd*	Fas (TNFRSF6)-associated via death domain	1.20	0.004
ILMN_2745954	NM_026269.1	*1110007L15Rik*	RIKEN cDNA 1110007L15 gene	1.20	0.020

Eleven separate primary tumors from 11 A-I Tg$^{+/-}$ or A-I KO mice were subjected to differential gene expression analyses. B16F10L melanoma homograft grown in C57BL/6 mice deficient in apoA-I (A-I KO) or expressing human apoA-I (A-I Tg$^{+/-}$) were resected 7 days after inoculation and processed for RNA, microarray and data analysis as described in Methods.

Supplementary Table 3A: Genes downregulated in primary melanoma tumors from A-I Tg mice relative to A-I KO mice mapped to molecular & cellular functions in ingenuity pathway analysis

Lipid Metabolism	Small Molecules Biochemistry	Vitamin & Mingeral Metabolism	Cell Cycle	Protein Synthesis	Cell Morphology	Cellular Assembly & Organization	Cellular Development	Cellular Function & Maintenance
Cyp51a1	*Cyp51a1*	*Cyp51a1*	*Cdc20*	*Apex1*	*Adamts1*	*Cdc20*	*Adamts1*	*Dicer1*
Dhcr24	*Dhcr24*	*Dhcr24*	*Dicer1*	*Cirbp*	*Dicer1*	*Dhx9*	*Cirbp*	*Fadd*
Elovl1	*Elovl1*	*Hsd17b7*	*Fadd*	*Dhx9*	*Fadd*	*Dicer1*	*Dicer1*	*Gorasp2*
Hsd17b7	*Fbp2*	*Insig1*	*Gorasp2*	*Dicer1*	*Mbp*	*Gorasp2*	*Fadd*	*Mbp*
Insig1	*Gale*	*Mpdu1*	*Slc19a1*	*Eif5a*	*Mest*	*Lims2*	*Get4*	*Stk25*
Mpdu1	*Hsd17b7*	*Msmo1*		*Fadd*	*Srsf5*	*Mbp*	*Mbp*	*Stx18*
Msmo1	*Insig1*	*Mvd*		*Mybbp1a*	*Tmpo*	*Mpv17*	*Nle1*	
Mvd	*Mpdu1*	*Slc19a1*		*Nup107*		*Nup107*		
Nme4	*Msmo1*	*Sqle*		*Vimp*		*Srsf5*		
Phgdh	*Mvd*	*Stard4*		*Wars*		*Stk25*		
Sqle	*Nme4*					*Stx18*		
Stard4	*Phgdh*					*Tmpo*		
	Slc19a1					*Vti1a*		
	Sqle							
	Srm							
	Stard4							

Supplementary Table 3B: Genes down-regulated in primary melanoma tumors from A-I Tg mice relative to A-I KO mice mapped to canonical pathways in ingenuity pathway analysis

Superpathway of Cholesterol Biosynthesis	Cholesterol Biosynthesis I	Cholesterol Biosynthesis II	Cholesterol Biosynthesis III	Zymosterol Biosynthesis
Cyp51a1	*Cyp51a1*	*Cyp51a1*	*Cyp51a1*	*Cyp51a1*
Dhcr24	*Dhcr24*	*Dhcr24*	*Dhcr24*	*Hsd17b7*
Hsd17b7	*Hsd17b7*	*Hsd17b7*	*Hsd17b7*	*Msmo1*
Msmo1	*Msmo1*	*Msmo1*	*Msmo1*	
Mvd	*Sqle*	*Sqle*	*Sqle*	
Sqle				

List of genes downregulated in primary melanoma tumors from A-I Tg mice relative to A-I KO mice mapped to (A) molecular & cellular functions or (B) canonical pathways in Ingenuity Pathway Analysis. Genes significantly associated with each process are listed.
